# PCR Conditions for the Detection of Molecular Markers Associated with Blackleg (*Leptosphaeria* spp.) Resistance in Rapeseed (*Brassica napus* L.)

**DOI:** 10.3390/ijms27146146

**Published:** 2026-07-09

**Authors:** Tomasz Jamruszka, Ewa Starosta, Justyna Szwarc, Magdalena Grynia, Janetta Niemann

**Affiliations:** 1 Department of Genetics and Plant Breeding, Poznań University of Life Sciences, Dojazd 11, 60-632 Poznań, Poland; tomasz.jamruszka@up.poznan.pl (T.J.); ewa.starosta@up.poznan.pl (E.S.); justyna.szwarc@up.poznan.pl (J.S.); 2 Strzelce Plant Breeding Ltd. IHAR Group, Borowo Department, Borowo 35, 64-020 Czempiń, Poland; m_grynia@hr-strzelce.pl

**Keywords:** *Brassica napus*, blackleg resistance, *Leptosphaeria* spp., DArTseq, molecular markers, SNP-type markers, SilicoDArT-type markers, polymerase chain reaction

## Abstract

Blackleg disease, caused by *Leptosphaeria* spp. fungi, is a major contributor to significant global yield losses in *Brassica napus*. Thus, selecting resistant plants using molecular markers linked to resistance loci is a common mitigation strategy. The latter, however, faces a challenge as pathogen virulence can overcome host resistance. This necessitates the identification of superior resistant genotypes through the use of numerous novel molecular markers and simplified detection methods to accelerate breeding programs. Based on our previous work, this study evaluated and verified molecular markers linked to blackleg disease resistance. A crucial finding is the identification of polymorphisms within SilicoDArT-type marker sequences that directly confer resistance. We also provide primer sequences for conventional PCR-based detection of SNP-type and SilicoDArT-type markers and a modified PCR protocol to enhance SNP detection efficiency. These validated markers and optimized PCR conditions are expected to significantly aid plant breeders in developing new, more resistant rapeseed varieties.

## 1. Introduction

Rapeseed (*Brassica napus* L.) is one of the most important oilseed crops in the world. In 2024, its production reached 85.26 million metric tons, and the total volume of rapeseed oil production amounted to about 32.7 million metric tons [1]. Poland, being one of the top producers in Europe, in the entire 2023/2024 season supplied 1555 thousand tons of rapeseed oil [2]. Large increase in rapeseed acreage and the deployment of ‘double zero’ varieties resulted in higher yields and improved oil quality [3]. However, over the next few years, the growth in rapeseed production is expected to decelerate [4]. Global demand for rapeseed requires vast agricultural areas and increased crop productivity [5]. Sadly, rapeseed crop cultivation relies strongly on global prices, as farmers often choose crop species based on market value and profitability [6]. Furthermore, shrinking resources and urbanization significantly reduces the cropping potential of rapeseed [7].

Rapeseed is vulnerable to abiotic, as well as biotic stresses. Salt stress, dehydration, waterlogging, extreme temperatures, and metal toxicity pose a particular threat [8]. On the other hand, yield losses caused by various biotic factors, such as insects, bacteria, viruses, and fungi, which affect this plant at different growth stages, reach 19.9% [9,10]. One of the most economically important rapeseed diseases is stem cancer, also known as blackleg. It is caused by a fungal complex consisting of *Leptosphaeria maculans* and *Leptosphaeria biglobosa* species [11]. The greatest damage to the crop is caused by *L. maculans*. It can infect the plant at all stages of growth. The initial symptom is the appearance of gray lesions on cotyledons and lower leaves. As the disease advances, basal stem canker develops, with lesions observed on upper stems, leaves, and siliques [12].

Plant protection methods such as crop rotation, soil management, or biocontrol are often not efficient in rapeseed cultivation. Therefore, chemical plant protection is the most commonly used method for pest and disease control [13]. However, given the European and Polish agricultural regulations and the concept of a ‘necessary minimum’, the use of such measures needs to be reduced [14]. The development of resistant cultivars has become one of the major directions in modern plant breeding and agriculture. Unfortunately, the conventional strategy for resistance breeding is slow and inefficient [15]. *L. maculans* populations can adapt and mutate at a rapid pace. Furthermore, continuous monoculture of rapeseed encourages the expansion of the pathogen, causing considerable yield losses [16]. This disease is responsible for at least 10% of annual losses worldwide; in extreme cases, the yield loss amounts to 90% [17,18]. In Poland, it was reported that the yearly loss reaches 10–40% [19]. Genetic resistance plays a key role in the deployment of resistant cultivars of rapeseed, particularly in relation to major resistance genes (*R*-genes). Molecular recognition between the qualitative *R*-genes in the host plant and the pathogen’s avirulence genes (*Avr*) is the basis for genetically mediated resistance mechanisms. Such a relation initiates a cascade of reactions within the host cells to effect immunity [20]. To date, 18 blackleg resistance genes have been identified in several *Brassica* species. Five of them (*Rlm2*, *Rlm4*, *Rlm7*, *Rlm9* and *LepR3*) were cloned and functionally assessed [21]. This form of resistance is most prominent in the early stages of plant development [22]. *R*-genes have been widely deployed in commercial cultivars of rapeseed and have been stated to be effective as a crop protection strategy. However, the intensive use of such a solution in combination with monoculture placed a strong selection pressure, causing the pathogen to overcome *R*-mediated resistance. It was reported that qualitative resistance can be broken within 3 years of cultivar deployment. In the long term, plant resistance based solely on *R*-genes is inefficient [23].

Rapeseed resistance mediated quantitatively by several genes (QR) with various genetic effects has been reported to prolong and enhance the effectiveness of qualitative resistance [24]. However, the understanding of underlaying QR mechanism is still poor [25]. The number of genes involved in plant immunity is mostly unknown, even though several QR loci have been identified [26]. The advancement of molecular resources facilitates the development of molecular markers that are associated with disease resistance and the identification of novel blackleg resistance genes [27]. Molecular markers can be used in place of the laborious and time-consuming method of phenotyping when screening for resistance. This way, genetic gains for controlling blackleg can be accelerated through speed breeding [28]. Due to the dual specificity of epistasis of *Avr* genes, phenotyping rapeseed for major resistance genes using differential sets of *L. maculans* is challenging. Some of the *Avr* genes are recognized by more than one resistance gene [29]. Furthermore, several known *R*-genes are alleles of the same locus [30].

Next-generation sequencing (NGS) systems have quickly become available and relatively affordable for identifying QTLs. In addition, parallel advances in computational science and statistical genetics allowed the association of NGS-derived polymorphisms with traits of interest for the establishment of new molecular markers [31]. The latter appear to be of varied nature, including insertions and deletions (indels) and single-nucleotide polymorphisms (SNPs) [32,33]. However, the main challenge is to incorporate identified markers in marker-assisted selection (MAS) breeding. One reason is that relatively few SNP markers have been validated for rapeseed blackleg resistance screening through MAS in small-scale laboratories due to their characteristics. Single-base polymorphisms are difficult to detect using non-sequencing methods in a reliable manner. There is a limited option for an affordable SNP system that does not require specialized and cost-intensive laboratory equipment. Several methods designed to genotype SNPs have been developed over the years. One of the first was the TaqMan system (Applied Biosystems, Inc., Waltham, MA, USA), based on allele-specific fluorescent probes utilizing the real-time PCR technique. KASP^TM^ (Kompetitive Allele Specific PCR) has become one of the top SNP genotyping platforms developed by KBioscience Ltd. (Guildford, UK). Similarly, it is an allele-specific genotyping technology, based on extension of oligonucleotides and the detection of signal generated by fluorescence energy transfer (FRET). Another method, rhAmp, utilizes RNase H2 to improve the efficiency and specificity of PCR. These approaches require equipment designed for fluorescent signal detection [34]. A simple, gel-based method called Amplification Refractory Mutation System (ARMS) has also been reported, utilizing PCR with an additional nucleotide mismatch at the 3′ primer end. This approach has been widely used for allele discrimination. However, such modifications are not consistently effective [35].

This work focused on assessment and validation of molecular markers (SNP-type and SilicoDArT-type) linked to blackleg disease resistance reported in our previous study [27]. Furthermore, we identify polymorphisms present within SilicoDArT-type marker sequences directly involved in the resistance. Moreover, we propose primer sequences for the identification of markers of both types using PCR, as well as a modification of the PCR protocol for efficient SNP detection. As a result, reported markers together with presented PCR conditions may be of use to plant breeders working on the development of new rapeseed varieties.

## 2. Results

### 2.1. Phenotyping

Phenotypic assessment of the level of rapeseed DH lines’ resistance to blackleg was analyzed. The disease symptoms were evaluated using a 10-point severity scale, where 0 represents no visible symptoms and 9 indicates severely damaged plants with numerous lesions and pycnidia, according to Jędryczka (2006) [36]. Rapeseed DH lines from the lower-resistance group were infected in varying degrees, ranging between 3 and 7 with an average of 3.77. In contrast, rapeseed DH lines attributed to the higher-resistance group were characterized by little to no blackleg disease symptoms, with an average of 0.43 and the highest score of infection severity reaching 0.6 (Table 1). The difference in the resistance between the two groups was statistically significant (*p* < 0.001), based on the Mann–Whitney test (normality assumptions were not met).

### 2.2. Identification of Polymorphisms and Design of PCR Primers

**Figure 1:**
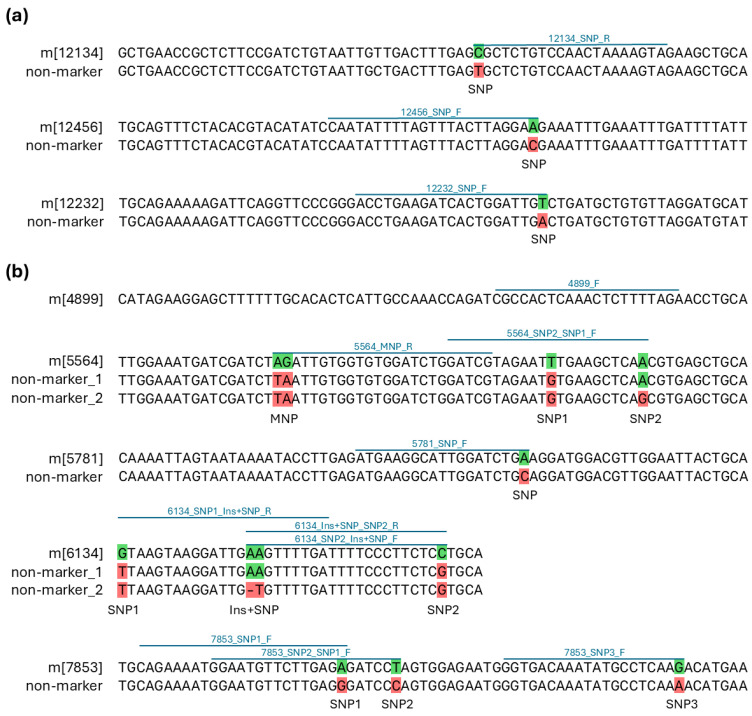
Alignment of single-nucleotide polymorphism (SNP)-type (**a**) and SilicoDArT-type (**b**) marker sequences with non-marker sequences identified previously [27] or found using BLAST tool, accessible on the BnIR website, respectively. Polymorphic variants present within markers are highlighted in green, whereas the alternative non-marker variants are highlighted in red. Blue lines indicate the fragments of marker sequences based on which the polymorphism-spanning primers were designed; corresponding marker-spanning primer names are also shown. MNP—Multiple-nucleotide polymorphism; Ins—Insertion.

Out of the fifteen markers previously characterized as linked to blackleg resistance [27], eight yielded specific and efficient PCR product amplification. Three of these eight markers were SNP-type, i.e., m[12134], m[12456] and m[12232], for which the positions of the SNP marker nucleotides and their respective non-marker nucleotides were also previously reported (Figure 1a) [27]. The remaining five markers, i.e., m[4899], m[5564], m[5781], m[6134], and m[7853], were SilicoDArT-type markers (Figure 1b). Analysis of the latter began by searching for alternative non-marker sequences in available rapeseed reference genomes using the BLAST tool, accessible on the BnIR website, in order to identify potential polymorphisms. For the marker m[4899], no alternative sequences were found. Two SNPs and one multiple-nucleotide polymorphism (MNP) with two subsequent SNPs were identified in m[5564]. One SNP was found in m[5781]. Marker m[6134] consisted of two SNPs and a complex polymorphism comprising a neighboring insertion and an SNP, which were further analyzed jointly. Finally, three SNPs were detected in m[7853] (Figure 1b).

Consequently, PCR primer pairs with one primer spanning the identified polymorphism (or two polymorphisms at once) were designed (Table 2). To enable SNP discrimination, the respective complementary nucleotide was placed on the 3′ end of the primer and its penultimate nucleotide was changed to a non-complementary one [35] to increase the difference between initiation efficiency of PCR in the presence of marker and non-marker sequences (for more information, see Section 4). The fragments of markers used as a template for primer design are indicated in Figure 1. Designed primer sequences are listed in Table 2. The standard procedure of temperature gradient PCR was used to determine the optimal PCR annealing temperature for each primer pair (Table 2, Supplementary Figure S1). Among all 13 primer pairs tested, only one pair (6134_SNP1_Ins+SNP_R and 6134_F) for marker m[6134] did not yield a PCR product. This pair was excluded from further analysis (Table 2).

### 2.3. Genotyping of Rapeseed DH Lines

Consequently, 12 PCR primer pairs were assessed for their differentiating ability between rapeseed DH lines with higher and lower resistance to blackleg.

**Figure 2:**
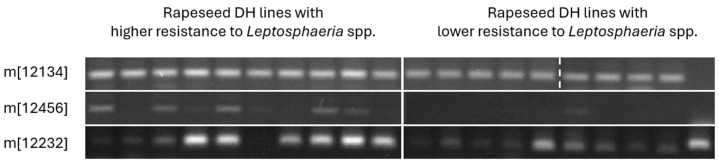
Gel electrophoresis results of PCR products for identification of SNP-type markers: m[12134] (primers: 12134_SNP_R and 12134_F), m[12456] (primers: 12456_SNP_F and 12456_R), and m[12232] (primers: 12232_SNP_F and 12232_R) in the DNA of rapeseed plants exhibiting higher (DH lines 1–10) and lower (DH lines 11–20) resistance to *Leptosphaeria* spp. Predicted PCR product sizes: 153 bp, 215 bp, and 202 bp, respectively. For marker m[12134], two separate agarose gel images were merged and are divided by a vertical dashed line. The uncropped, original gel images are provided as Supplementary Figures S2 and S3.

Primers designed for SNP-type markers, m[12134], m[12456] and m[12232], facilitated more efficient amplification of PCR product amplified on the basis of DNA samples extracted from certain plants exhibiting higher resistance, in contrast to the group of plants with lower resistance. However, the difference in band intensity was much more pronounced in the case of marker m[12456] and m[122323] compared to m[12134]. For marker m[12456], four DH lines out of ten with higher resistance produced the most intense bands, whereas no bands or only weak bands were observed in plants with lower resistance. For marker m[12232], 5–6 and 1–2, the most intense bands were visible in both groups, respectively (Figure 2, Table 3).

**Figure 3:**
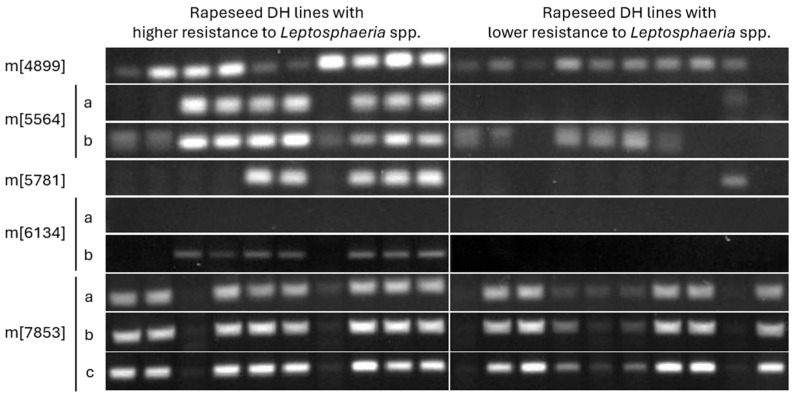
Gel electrophoresis results of PCR products for identification of SilicoDArT-type markers: m[4899] (primers: 4899_F and 4899_R), m[5564] (primers: a—5564_MNP_R and 5564_F; b—5564_SNP2_SNP1_F and 5564_R), m[5781] (primers: 5781_SNP_F and 5781_R), m[6134] (primers: a—6134_Ins+SNP_SNP2_R and 6134_F; b—6134_SNP2_Ins+SNP_F and 6134_R), and m[7853] (primers: a—7853_SNP1_F and 7853_R; b—7853_SNP2_SNP1_F and 7853_R; c—7853_SNP3_F and 7853_R) in the DNA of rapeseed plants exhibiting higher (DH lines 1–10) and lower (DH lines 11–20) resistance to *Leptosphaeria* spp. Predicted PCR product sizes: 216 bp, 188 bp, 191 bp, 203 bp, 196 bp, 186 bp, 324 bp, 316 bp, and 284 bp, respectively. The uncropped, original gel images are provided as Supplementary Figures S4–S9.

Primers designed for the detection of SilicoDArT-type marker m[4899] consistently produced an amplification product across nearly all analyzed DNA samples. However, a noticeable increase in the intensity of seven bands on the agarose gel exhibited a strong correlation with the group of plants that demonstrate enhanced resistance (Figure 3, Table 3).

Two primer pairs, designed to detect two fragments of the SilicoDArT-type marker m[5564] (Figure 1), demonstrated differential PCR amplification efficiency between rapeseed samples exhibiting higher and lower resistance to blackleg. In both instances, a majority of plants, i.e., 6–7, with higher resistance consistently showed the most intense bands (Figure 3, Table 3). Nevertheless, the primer pair designed for the simultaneous detection of two separate SNPs (SNP1 and SNP2) performed less optimally (Figure 3). This was evidenced by faint bands in some samples, in contrast to the primer pair designed to identify a multiple-nucleotide polymorphism (MNP) consisting of two consecutive SNPs, where almost no faint bands were visible (Figure 3).

PCR with primers designed to detect the adenine (A) nucleotide in the SilicoDArT-type marker m[5781] showed correlation with plant resistance. Stronger bands were observed in five out of ten plants with increased resistance (Figure 3, Table 3).

Of the three primer pairs designed to identify SilicoDArT-type marker m[6134], two successfully amplified a PCR product (Figure 3, Table 3). Both pairs targeted the same polymorphisms: one consisting of an insertion and SNP (Ins+SNP), and the second being a single SNP (referred to here as SNP2). However, the primers in each pair that spanned the markers had differing orientations, leading to a different polymorphism at their 3′ ends (Figure 1). Unfortunately, the first primer pair, which was designed to target the Ins+SNP at its 3′ end, produced a non-specific PCR product over 500 bp (Supplementary Figure S6). This was significantly larger than the expected size of 196 bp (Figure 3). In contrast, the second primer pair, with SNP2 at its 3′ end, yielded a specific product whose presence correlated with increased plant resistance: seven bands were observed in this group, whereas none were visible in the less resistant group (Figure 3, Table 3).

Three primer pairs designed for the identification of the SilicoDArT-type marker m[7853] exhibited almost the same banding pattern in all tested plants. However, this pattern was only weakly correlated with blackleg resistance: eight plants from the higher-resistance group and five plants from the lower-resistance group produced the most intense bands, respectively (Figure 3, Table 3).

### 2.4. Confirmation of Plant Genotype

Marker *loci* in selected plants, including those that yielded the most efficient PCR product amplification and those that yielded less efficient or no product, were analyzed by Sanger sequencing. Unfortunately, for two markers, m[6134] and m[12134], the amplified PCR products were off-target amplicons. For the remaining six markers, the product containing the marker *locus* sequence was successfully amplified.

**Figure 4:**
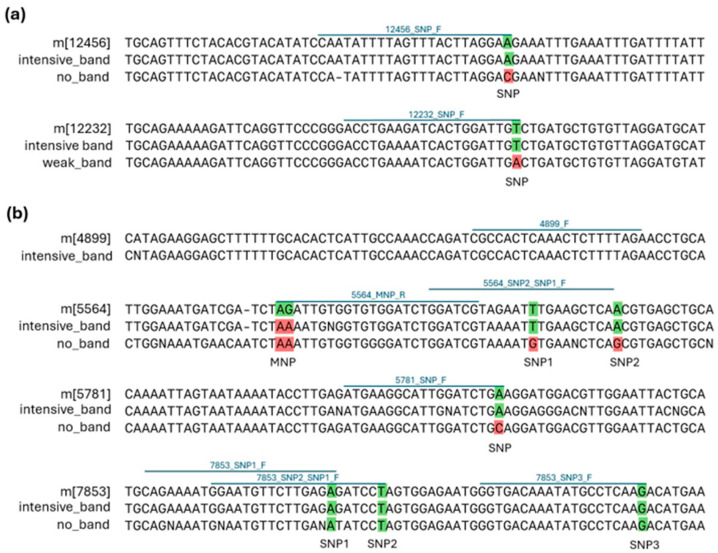
Alignment of SNP-type (**a**) and SilicoDArT-type (**b**) marker sequences with sequences detected in plants that generated the most intense bands and weak/no bands during marker identification. Marker variants of polymorphisms are highlighted in green, whereas the alternative non-marker variants are highlighted in red. Blue lines indicate the fragments of marker sequences based on which the polymorphism-spanning primers were designed; corresponding marker-spanning primer names are also shown. MNP—Multiple-nucleotide polymorphism; Ins—insertion. Nucleotides of unidentified nature during sequencing are assigned as N.

The analyses confirmed that the target SNPs within the SNP-type markers, specifically m[12456] and m[12232], were present in the plants that generated the most intense bands on agarose gel and absent in the plants generating weak or no bands. A similar pattern was observed for the SilicoDArT-type marker m[5781], which initially had an SNP detected. The marker sequence m[4899] was found in one of the plants that exhibited efficient PCR during its identification. Furthermore, it was confirmed that the two identified SNP alleles, T (SNP1) and A (SNP2), in marker m[5564] are present in plants that generated more PCR product, while plants generating weak product possess alternative alleles. However, in the latter marker, the presence of an MNP was not confirmed; instead, an additional SNP was found along with previously unidentified polymorphisms, including an indel. The marker sequence m[7853] was found in both types of analyzed plants (Figure 4). Fluorograms are presented in Supplementary Figure S10.

### 2.5. Comparative Trial of DNA Profiling Using Reference Control Lines

**Figure 5:**
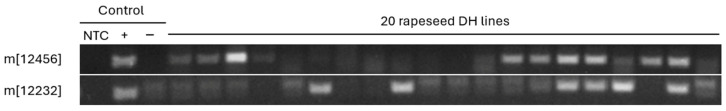
Gel electrophoresis results of PCR comparative trial identification of SNP-type markers: m[12456] (primers: 12456_SNP_F and 12456_R) and m[12232] (primers: 12232_SNP_F and 12232_R) in the DNA of 20 rapeseed DH lines. NTC: No template control; “+”: positive control (DNA of a DH line possessing the marker); “−“: negative control (DNA of a DH line lacking the marker). Expected product sizes: 215 bp and 202 bp, respectively. The uncropped, original gel images are provided as Supplementary Figures S11 and S12.

**Figure 6:**
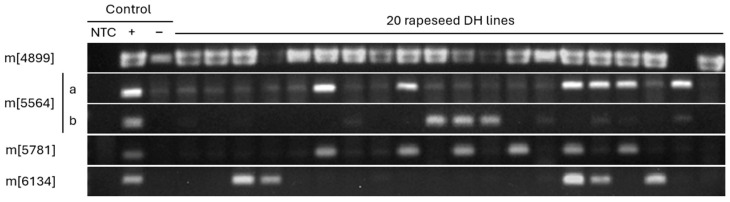
Gel electrophoresis results of PCR comparative trial identification of SilicoDArT-type markers: m[4899] (primers: 4899_F and 4899_R), m[5564] (primers: a—5564_MNP_R and 5564_F; b—5564_SNP2_SNP1_F and 5564_R), m[5781] (primers: 5781_SNP_F and 5781_R), m[6134] (primers: 6134_SNP2_Ins+SNP_F and 6134_R) in the DNA of 20 rapeseed DH lines. NTC: No template control; “+”: positive control (DNA of a DH line possessing the marker); “−“: negative control (DNA of a DH line lacking the marker). Expected product sizes: 216 bp, 188 bp, 191 bp, 203 bp and 186 bp, respectively. The uncropped, original gel images are provided as Supplementary Figures S11–S15.

A preliminary validation of the PCR primer pairs that distinguished resistance levels (Section 2.3) was performed using a panel of 20 rapeseed DH lines (a new set, distinct from the one analyzed in Section 2.3). Three control samples were included: a no-template control (NTC), as well as positive and negative controls represented by genotypes sequenced in the previous stage of the study, in which the presence or absence of the target polymorphism had been confirmed. The positive control produced the expected amplification product, whereas no specific product was observed for the negative control. The obtained banding patterns confirmed that the primers generated bands of varying intensity, indicating the presence or absence of a specific SNP-type (Figure 5) or SilicoDArT-type (Figure 6) marker.

## 3. Discussion

Blackleg stands as one of the most crucial fungal diseases affecting *Brassica* species. Improving rapeseed blackleg resistance is therefore essential for protecting crops worldwide [11]. In this context, the marker-assisted selection (MAS) approach evaluated in our study provides a powerful tool for accelerating breeding programs. While traditional markers, such as RFLPs, AFLPs, or RAPDs, have historically been used, their application is frequently limited by the risk of recombination breaking the linkage between the marker and the resistance trait in subsequent generations [37]. However, recent years have seen a rapid rise in the identification of molecular markers with precisely defined sequences and associated genes that ensure high diagnostic reliability across generations without the risk of linkage breakdown. This is due to, *inter alia*, increasingly frequent use of next-generation sequencing (NGS) techniques [31]. One of the NGS-based techniques used to identify DNA markers that were analyzed in this work is Diversity Arrays Technology sequencing (DArTseq), which utilizes a genome complexity reduction step, allowing the analyses to focus on transcriptionally active regions of the genome [38].

The utilization of two plant groups with contrasting levels of resistance to *Leptosphaeria* spp. in this study enabled the subsequent validation of the designed primers. Analyzing ten plants per group allowed for correlating their performance with plant resistance. The significance of this analysis was further enhanced by the fact that the analyzed markers had already been previously correlated with the trait under study [27], and the aforementioned PCR assays with the designed primers were only intended to confirm this (Figure 2 and Figure 3). However, incorporating a more diverse germplasm represents a crucial direction for future breeding programs to expand upon these findings.

The degree of resistance in both groups differed significantly (*p* < 0.001). However, while the mean values in both groups indicated a relatively high level of resistance overall, they were not vastly different from each other, with a difference of 3.34 on a 10-point scale (Table 1). It should be noted that the field evaluation of blackleg resistance in the DH population was conducted under meteorological conditions that, while favorable for winter oilseed rape growth, were suboptimal for natural infection and symptom development of *Leptosphaeria maculans*. In particular, June and July 2023 were dry and warm, and these conditions contrast with the humid weather typically required for efficient infection and lesion progression [39]. As a result, the disease pressure in the field was likely lower than expected, which might have further implications for the association analyses [40]. These constraints could have diminished the contrast required for robust genotype–phenotype associations, reducing the GWAS power as quantitative resistance loci may remain undetected, due to strong genotype–environment interaction effects [41]. Nevertheless, the loci detected despite low disease pressure may still represent strong, stable resistance factors.

Despite the low level of resistance variation between individuals, differentiated PCR results for the analyzed markers were obtained in both groups. This may suggest the high utility of the analyzed markers in detecting germplasm with the highest potential as a blackleg genetic resistance donor, which is particularly desirable in plant breeding programs.

DArTseq identifies highly polymorphic DNA markers, which are typically 69 base pairs long (with some exceptions) [42]. However, polymorphic nucleotides usually constitute only a small part of the marker’s sequence. For SNP-type markers, only a single SNP is identified. Our sequencing analyses confirmed the SNPs identified during the NGS stage for markers m[12456] and m[12232] (Figure 4). On the other hand, SilicoDArT-type markers can exhibit either a similar or a more complex structure [38]. Our current study made a comparable observation: among the polymorphisms found within SilicoDArT-type markers, we predominantly detected SNPs, one or more dispersed along a marker sequence, but also indels (Figure 4). Analyzed SilicoDArT-type markers possessed different combinations of these polymorphisms, while marker m[5781] had only a single SNP (Figure 4). Its original classification as a SilicoDArT-type marker reflects the underlying technical differences in data processing pipelines rather than a fundamental biological divergence [38]. Moreover, the correlation analyses of the identified polymorphisms with rapeseed resistance to *Leptosphaeria* spp. conducted using PCR (Figure 3) and sequencing results (Figure 4) suggest that the structures of SilicoDArT-type markers were mostly correctly characterized.

DArTseq, together with other high-throughput technologies, has enabled the generation of a greater number of molecular markers. While these techniques are suitable for large-scale studies, they are often not economical for routine applications, such as plant screening in smaller laboratories. Consequently, the detection of markers in such laboratories is typically based on PCR techniques. Historically, resource-limited breeding programs relied on marker systems that do not require prior sequence information such as RAPD and AFLP [43,44,45,46,47,48]. Over time, this shifted toward a constantly expanding pool of sequence-characterized markers detectable via conventional PCR and agarose gel electrophoresis, including SCAR and SSR markers [44,49,50,51]. The utility of PCR-based markers for disease resistance dissection has been demonstrated in several studies. For instance, recent work by [52] highlights the successful development of novel clubroot resistance marker BraSSR45619_E4, whose stability and reliability were confirmed in a *B. rapa* population. The potential of SSR markers linked to clubroot resistance for large-scale identification has also been demonstrated for *Brassica* interspecific hybrids [53]. Furthermore, the accuracy and credibility of other PCR-based markers, i.e., CAPS and SCAR markers, showing linkage to the *Rlm6* blackleg resistance gene was proved in a *Brassica* backcross breeding program [51].

However, due to the length requiring high resolution, SNP markers belonging to this group are more frequently detected using techniques based on quantitative PCR (qPCR). Systems like TaqMan, KASP, and HRM are excellent for high-throughput genotyping. Their main advantage is that they eliminate the need for post-PCR gel electrophoresis, which saves laboratory time and reduces contamination risks [54]. However, these methods have significant financial and infrastructural limitations. They require expensive real-time PCR instruments, specialized software, and costly fluorescent probes or specific master mixes [55]. For smaller breeding stations with limited budgets, these costs are often too high. Our conventional PCR method directly addresses these limitations. Admittedly, this approach has a drawback: it requires subsequent agarose gel electrophoresis, making it more labor-intensive and slower than automated KASP or TaqMan platforms. However, its primary advantage is high accessibility. It requires only standard thermal cyclers and basic gel documentation systems to deliver clear, reliable presence-or-absence allele scoring. Therefore, this validated methodology provides an excellent, low-cost alternative for targeted marker-assisted selection in resource-limited environments [56,57].

When directly compared, PCR-based marker systems such as AS-PCR, SCAR, SSR, and CAPS differ significantly in their polymorphism detection capabilities, operational complexity, and developmental costs. While these conventional systems remain highly valuable for population analyses and robust allele discrimination, their deployment in routine breeding operations can be limited by labor-intensive scoring or the need for multi-step enzymatic digestions [58,59,60,61,62,63]. In this study, conventional PCR was chosen for the identification of molecular markers. As a fundamental technique in molecular biology, widely utilized in various types of laboratories, including those at plant breeding stations, the results of this research will be broadly applicable for daily plant selection [37]. However, PCR has certain limitations. Unlike other techniques for detecting specific markers, its ability to detect unknown mutations or variations is restricted, as it requires prior knowledge of the target sequence [64]. Furthermore, preparing a reliable PCR analysis requires fulfilling numerous conditions. The latter are of paramount importance in the described analyses, where the presence or absence of a marker is evaluated visually based on band intensity, which—due to poorer sample quality—may not reflect the actual concentration of the DNA template. Band intensity may be affected by DNA quality, the presence of contamination and PCR inhibitors, and differences in template concentration. Additionally, variability may arise during gel electrophoresis and visualization. Improper gel preparation, sample loading errors, and differences in DNA staining or imaging conditions may affect band intensity and interpretation [57]. This reliance on relative band intensity as a diagnostic criterion introduces a degree of subjectivity, as visual scoring is semi-quantitative and can lead to misinterpretations. For instance, partial PCR inhibition might be mistaken for a true negative or an alternative genotype, while minor non-specific amplification could mimic a weak positive signal, reducing the diagnostic certainty required for high-throughput screening. Thus, to minimize genotyping errors, DNA quality should be assessed prior to PCR, DNA concentrations should be normalized across samples, and all ambiguous genotypes should be verified through independent PCRs. Fortunately, an increasing number of laboratories, especially those that routinely perform DNA analysis, now possess automatic DNA isolation devices, which often reduces the issue of varying concentrations and purity. Therefore, to ensure the highest possible repeatability of the results, some PCR conditions were checked in this study, including the annealing temperature (Table 2) and the DNA polymerase suitable for the detected SNP or non-SNPs (Figure 1). Furthermore, utilizing control plants that either contain or lack specific markers could facilitate the evaluation of these markers’ presence in DNA samples, thereby improving the quality of successive PCR analyses from the very beginning (Figure 5 and Figure 6).

One of the primary challenges associated with using PCR for molecular marker identification is the technique’s limited sensitivity toward SNPs. Using standard protocols for such short polymorphisms in a DNA sequence does not allow for distinguishing their variants in different DNA samples. To overcome these difficulties, protocol modifications are essential [65]. In this study, we employed two of these, namely primer sequence changes and the use of a specific polymerase. Primer pair sequences for both types of markers, SilicoDArT-type and SNP-type, were designed with one primer spanning the marker and the second neighboring it (Table 2). Primers designed to attach to potential polymorphisms had the corresponding complementary nucleotide or nucleotides at their 3′ end (Figure 1, Table 2), crucial for the initiation of synthesis of the DNA strand during PCR [66]. To increase the difference in PCR initiation efficiency between the marker-containing SNP sequence and its alternative, non-marker sequence, the second nucleotide at the 3′ end of the spanning primer was changed to be non-complementary to both the marker-containing and non-marker sequences. This resulted in a single non-complementary nucleotide at the penultimate position for the marker sequence, and non-complementary nucleotides at both the ultimate and penultimate positions for the non-marker sequence (Figure 1, Table 2). This method, known as amplification-refractory mutation system (ARMS), is used in many scientific fields, including plant marker-assisted selection [67,68]. It is a highly effective cost-efficient method for SNP genotyping. The technique requires minimal laboratory infrastructure while providing an accessible approach for the detection of short DNA polymorphisms. The sequencing results showed that using ARMS was useful for identifying SNPs in this work as well, since almost all the designed primers had an SNP at the 3′ end (Figure 1). To enhance the effectiveness of the PCR protocol during SNP identification, we also utilized a DNA polymerase lacking 3′ → 5′ proofreading exonuclease activity. This enzyme, unlike OptiTaq DNA polymerase used in this work for identification of non-SNPs, is unable to correct a mismatch at the 3′ end of the spanning primer when it binds to a non-marker sequence [69].

Highly optimized primer pairs were successfully designed for markers: m[12456] (primers 12456_SNP_F and 12456_R), m[12232] (primers 12232_SNP_F and 12232_R), m[4899] (primers 4899_F and 4899_R), m[5564] (primers 5564_SNP2_SNP1_F and 5564_R), m[5781] (primer 5781_SNP_F and 5781_R) (Table 2). These primers clearly differentiated the blackleg-resistant plant group, reflecting the previously established linkage between these markers and resistance to *Leptosphaeria* spp. [25] (Figure 2 and Figure 3). Moreover, their marker/non-marker allele differentiation was confirmed by sequencing (Figure 4). The sequencing results also verified that the polymorphism-spanning primers (primers that attach to the marker) were successfully designed in the correct position (Table 2, Figure 4).

For m[5564], as mentioned earlier, only a primer detecting two SNPs (pair 5564_SNP2_SNP1_F) was effective. The other primer (5564_MNP_R) detecting predicted MNP should be redesigned to better reflect the marker and non-marker alleles detected during Sanger sequencing, e.g., marker T and non-marker G, despite differentiating resistance based on band intensity (Table 2, Figure 1).

A weak and ambiguous correlation with resistance was observed during the identification of marker m[12134]. This was also the only marker where the designed primer pair (12134_SNP_R and 12134_F) yielded poorly distinguishable differences in band intensity (Figure 2). Furthermore, it was one of two markers for which Sanger sequencing failed due to a lack of a specific PCR product.

The second marker that failed sequencing was m[6134]. For this marker, one designed primer pair (6134_Ins+SNP_SNP2_R and 6134_F) produced an oversized product (Figure 3). This lack of the expected PCR product was not attributed to the analyzed polymorphism having no impact on resistance, but rather to other factors. One potential factor could be that both primers in this pair may also bind to other *B. napus* chromosomes, including chromosome A03 (Supplementary Figure S16). Additionally, undetected polymorphisms might be present in the binding site for the 6134_F primer (which binds upstream of the marker), potentially affecting the results. Interestingly, for the same marker, simply positioning a single SNP at the 3′ end of the primer in an alternative pair (6134_SNP2_Ins+SNP_F and 6134_R) yielded the expected product, thereby successfully differentiating plant resistance (Figure 1 and Figure 3).

A highly reproducible pattern of PCR product formation was observed for all three primer pairs examining potential polymorphisms in marker m[7853]. However, these PCR products showed almost no correlation with plant resistance (Figure 3). This lack of correlation was further confirmed by the absence of sequence differences between plants that generated a PCR product (band) and those that did not (Figure 4). This indicates that the sequences available in public databases were insufficient to identify the specific polymorphisms underlying the marker’s linkage to resistance.

Our study successfully designed and validated PCR primers and conditions for identifying several SNP-type and SilicoDArT-type molecular markers associated with blackleg resistance in rapeseed. Considering the ability to differentiate resistance and detect an expected marker alleles confirmed by sequencing, the best primer pairs were those designed for five markers: m[12456] (primer pair 12456_SNP_F and 12456_R), m[12232] (12232_SNP_F and 12232_R), m[4899] (4899_F and 4899_R), m[5564] (5564_SNP2_SNP1_F and 5564_R), and m[5781] (5781_SNP_F and 5781_R) (Table 4). The remaining primer pairs either exhibited insufficient differences in band intensity (12134_SNP_R and 12134_F), failed to confirm marker linkage with resistance (primer pairs for m[7853]), sequencing did not confirm their correlation between banding patterns and the presence of specific alleles (5564_MNP_R and 5564_F; primer pairs for m[7853]), or we were unable to sequence the marker *loci* (12134_SNP_R and 12134_F; 6134_SNP2_Ins+SNP_F and 6134_R) (Table 4).

## 4. Materials and Methods

### 4.1. Plant Material

Plant material consisted of 20 rapeseed doubled-haploid (DH) lines derived from Strzelce Plant Breeding Ltd. (Strzelce, Poland) IHAR Group in Borowo (Table 5).

An additional set of rapeseed genotypes from Strzelce Plant Breeding Ltd. (Strzelce, Poland) IHAR Group in Borowo was included for primer pair validation (Table 6).

### 4.2. Field Assessment

All DH lines were assessed for blackleg resistance in field conditions. The plants were cultivated in experimental fields of Strzelce Plant Breeding Ltd. (Strzelce, Poland), IHAR Group, in Borowo. The planting was carried out in mid-August 2022. The experimental design involved the cultivation of rapeseed DH lines in plots measuring 10 m^2^, with a sowing density of 60 seeds per 1 square meter. Subsequently, they were evaluated in the BBCH 70–89 phase, using a 0–9 symptom severity scale, described by Jędryczka [36]. Score 0 was unequivocal with no visible disease symptoms, while score 9 indicates severely damaged plants with numerous lesions with pycnidia. Ten plants per plot were assessed. A detailed description of the criteria used during this evaluation was provided by Starosta et al. [27].

The meteorological conditions during the 2022–2023 growing season were generally favorable for winter oilseed rape growth and development. In Wielkopolska voivodeship, where the experimental fields are located, the mean air temperature in 2022 and 2023 was 10.6 °C and 10.8 °C, respectively [70,71]. The mean precipitation rate was 414.6 mm in 2022 and 710.5 mm in 2023. June and July 2023, which were crucial months for the disease occurrence assessment, were very dry and warm with total rainfall of 38.7 mm and 61.7 mm, and mean temperature of 19.4 °C and 20.5 °C [70,71].

### 4.3. DNA Extraction

Whole-genomic DNA was extracted from 7-day-old seedlings using the Genomic Mini AX Plant kit (A&A Biotechnology, Gdańsk, Poland). The concentration and purity of the isolated DNA were assessed using spectrophotometer DeNovix DS-11 (DeNovix Inc., Wilmington, DE, USA). Samples with concentrations higher than 100 ng/µL and 260/280, 260/230 ratios of ~1.8 and ~2.0, respectively, were used for further analysis. Prepared DNA samples were diluted to a concentration of 30 ng/µL.

### 4.4. PCR Primer Design

To characterize the previously identified SilicoDArT-type markers [27], their sequences from the plus DNA strand were compared to the available rapeseed reference genomes using the BLAST+ 2.16.0 tool implemented via the SequenceServer interface [v2.0.0] accessible on the *Brassica napus* multi-omics information resource (BnIR) website (https://yanglab.hzau.edu.cn/BnIR, accessed on 1 September 2024). Subsequently, alignment to the resulting sequences using the CLUSTALW tool accessible on PRABI-GERLAND bioinformatics platform (https://npsa-pbil.ibcp.fr, accessed on 1 September 2024) enabled identification of putative polymorphisms in the analyzed marker sequences. In contrast to DArT-type markers, the positions of SNP marker nucleotides in SNP-type markers and their respective non-marker nucleotides were previously determined [27].

For each marker, polymerase chain reaction (PCR) primer pairs were designed with one primer spanning the identified polymorphism and the other complementary to the sequence neighboring the marker. All primers were designed according to commonly accepted criteria, considered important for their effectiveness, i.e., length 18–25 bp, melting temperature 50–60 °C, GC content 40–60%, and the presence or absence of unwanted interactions between primers in pairs. These features also guided the selection of forward (F) or reverse (R) primer orientation. Marker-spanning primers were designed to be complementary to at least one detected polymorphism at their 3′ end. In some cases, a single primer spanned more than one polymorphism. In the case of SNP identification, a polymorphic nucleotide position was also localized at the 3′ end of the respective primer. If the primer covered two SNPs, or an SNP together with another type of polymorphism, only one SNP was placed at the 3′ end. Additionally, to increase the difference in the efficiency of amplification initiation between marker and non-marker sequences containing varying SNP nucleotides, the penultimate nucleotide at the 3′ end of each SNP-spanning primer was replaced with an appropriate noncomplementary nucleotide according to Little (2001) [35]. Primers spanning the respective polymorphisms among markers were named as follows: *marker name*_*detected polymorphisms in order from the primer’s 3′ end*_*primer orientation (F or R).* Primers neighboring markers, paired with primers spanning markers during PCR, were named as follows: *marker name_primer orientation (F or R)*.

All primers used in this study were synthesized by the DNA Sequencing and Synthesis Facility (IBB PAN, Warsaw, Poland). Primers were supplied in lyophilized form. The primers were produced to be standard purity-grade, ethanol-precipitated, and suitable for PCR and sequencing. Upon delivery, the lyophilized primers were stored at 5–10 °C until resuspension. Primers were dissolved in nuclease-free, molecular-grade water to a stock concentration of 100 μM, according to the manufacturer’s instructions. Stock solutions were aliquoted and stored at −20 °C to prevent degradation. For PCR analysis, 10 μM working solutions were prepared from the stocks to minimize freeze–thaw cycles and ensure consistent primer performance across experiments.

The optimal PCR annealing temperature of each primer pair was determined using temperature gradient PCR. The predicted size of PCR products generated using the tested primer pairs was determined based on the sequence of the Darmor-*bzh* v4.1 reference genome.

### 4.5. Genotyping

Designed primer pairs were used to genotype analyzed rapeseed plants (Section 4.1) using PCR. The primer sequences were designed as described in the Section 4.4.

For the detection of non-SNPs using primers with complementary nucleotide(s) at 3′ end, a PCR procedure with OptiTaq DNA polymerase that possesses proof-reading activity was conducted. The PCR was performed in a total volume of 25 µL per sample and contained: 12.5 µL Color OptiTaq Master Mix (2× (EURx, Gdańsk, Poland), 1 µL forward (F) primer (10 μM), 1 µL reverse (R) primer (10 μM), 7.3 µL H_2_O, 3.2 µL DNA template (~96 ng). The reaction conditions were as follows: initial denaturation at 95 °C for 3 min, 40 cycles of amplification (denaturation at 95 °C for 30 s, annealing at specific temperature for 30 s, extension at 72 °C for 1 min), and final extension at 72 °C for 5 min.

For the detection of SNPs using primers with complementary nucleotides at the 3′ end, a modified PCR procedure with a polymerase lacking 3′ → 5′ proofreading exonuclease activity was conducted. The PCR was performed in total volume of 25 µL per sample and contained: 2.5 µL ThermoPol Reaction Buffer (10×) (New England Biolabs, Ipswich, MA, USA), 0.5 µL deoxynucleotide (dNTP) solution mix (10 mM), 1 µL forward (F) primer (10 μM), 1 µL reverse (R) primer (10 μM), 0.25 DeepVent (exo-) DNA Polymerase (New England Biolabs, Ipswich, MA, USA), 1.5 µL, MgSO_4_ (6 mM), 3.2 µL DNA template (~96 ng) and 15.05 µL H_2_O. The reaction conditions were as follows: initial denaturation at 95 °C for 5 min, 40 cycles of amplification (denaturation at 95 °C, annealing at specific temperature—Table 2—for 30 s, extension at 72 °C for 30 s), and final extension at 72 °C for 5 min.

All PCRs were carried out in Bio-Rad thermocycler C1000 Touch (Bio-Rad, Herkules, CA, USA).

For the visualization of DNA amplification products, the DNA fragments were separated in a 1% agarose gel, with the addition of Midori Green Advanced DNA (Nippon Genetics, Tokyo, Japan) stain (5 µL per 100 µL of agarose solution). The electrophoresis was conducted for 1 h at 110 V. The visualization and image capture were performed using a Molecular Imager^®^ Bio-Rad Gel Doc^TM^ XR+ supplemented by Image Lab^TM^ 5.2 Software (Bio-Rad, CA, USA). DNA molecular weight marker Perfect^TM^ 100 bp DNA Ladder (EURx, Gdańsk, Poland) was used to determine the approximate size of amplified DNA fragments. To improve the simultaneous visualization of amplification results across all 20 analyzed DH lines, images from samples located in two separate agarose gel rows were merged in certain cases.

### 4.6. Confirmation of Plant Genotype

The genotypes of plants that yielded efficient PCR product amplification (potential marker carriers) and those that yielded little/no product (potential non-marker carriers) were assessed. For this purpose, new primer pairs spanning marker sequences were designed (Supplementary Table S1). Their annealing temperatures were assessed through gradient PCR (Supplementary Figure S17) using a protocol for Deep Vent (exo-) DNA polymerase (described in the Section 4.5). Next, the designed primers were used in a PCR with DNA isolated from the genotypes that generated intensive bands and those that generated weaker or no bands during marker identification. This step aimed to select the genotypes that produced bands suitable for further sequencing (Supplementary Figure S18). For each marker, PCR products from two genotypes—one generating intensive bands and one weak/no bands (Table 7)—were analyzed by Sanger sequencing. The exception was m[4899], where no PCR product was generated from the plants that did not generate bands during identification, so only a sample from the intense band-generating plant was used (Table 7). Sanger sequencing was performed using the forward primer that was used to prepare PCR products (Supplementary Table S1).

## 5. Conclusions

In response to the global threat of blackleg disease, this research successfully developed and evaluated accessible conventional PCR primers for identifying key resistance markers in rapeseed, with primer pairs for five markers showing high effectiveness while others required further technical refinement. Notably, by employing modified primer structures and a specific DNA polymerase, we effectively overcame the challenge of differentiating short polymorphisms across plant groups. While this differentiation relies on comparing the relative intensity of amplification bands, such visual scoring remains susceptible to technical and experimental variation. To address this limitation, all critical steps in this study were carefully optimized and standardized to minimize potential sources of error. Consequently, this validated methodology provides an accessible, low-cost, and highly practical tool for daily plant selection in breeding programs.

## 6. Patent

The results described herein, concerning the identification of markers m[4899], m[5564], and m[5781], are part of Polish Patent Application No. P.452021.

## Figures and Tables

**Table 1:** Resistance of DH lines assessed using a 10-point severity scale (0—no visible symptoms, 9—severely damaged plants with numerous lesions and pycnidia).

Rapeseed DH Lines with Higher Resistance to *Leptosphaeria* spp.			Rapeseed DH Lines with Lower Resistance to *Leptosphaeria* spp.		
No.	Line ID	Symptom Severity (0–9 Scale)	No.	Line ID	Symptom Severity (0–9 Scale)
1.	DH2 0/21DH6720	0.5	11.	DH2 0/21DH6702	3.2
2.	DH2 0/21DH9455	0.6	12.	DH2 0/21DH6721	3.4
3.	DH2 0/21DH6762	0.0	13.	DH2 0/21DH6745	4.6
4.	DH2 0/21DH9440	0.6	14.	DH2 0/21DH6730	3.2
5.	DH21 0/21DH7414	0.6	15.	DH2 0/21DH6752	3.4
6.	DH21 0/21DH7433	0.6	16.	DH2 0/21DH9444	7.0
7.	DH2 0/21DH9525	0.4	17.	DH2 0/21DH6761	3.0
8.	DH22 0/21DH7477	0.0	18.	DH2 0/21DH9488	3.5
9.	DH22 0/21DH7481	0.5	19.	DH21 0/21DH7445	3.2
10.	DH20 0/21DH8950	0.5	20.	DH27 0/21DH7988	3.2

**Table 2:** List of primer pairs used for the PCR amplification of SNP-type and SilicoDArT-type marker fragments containing detected variants of polymorphisms.

Marker Name	Name of Spanning Primer	Sequence of Polymorphism-Spanning Primer (5′→3′)	Name of Neighboring Primer	Sequence of Marker-Neighboring Primer (5′→3′)	Expected Product Size [bp]	Annealing Temperature [°C]
m[12134]	12134_SNP_R	TACTTTTAGTTGGACAGAGAG	12134_F	CGTCCTATCTTTTCCATAGAG	153	50
m[12456]	12456_SNP_F	CAATATTTTAGTTTACTTAGGGA	12456_R	TGGAAAGCATTAAAGAAAA	215	50
m[12232]	12232_SNP_F	ACCTGAAGATCACTGGATTAT	12232_R	CCAAATAAATAAGTATCGAAGTTCA	202	50
m[4899]	4899_F	CGCCACTCAAACTCTTTTAG	4899_R	AGACCCATTATCGCCTTATC	216	58
m[5564]	5564_MNP_R	CGATCCAGATCCACACCACAATCT	5564_F	CTTGTAGTGTATAGGGGCTG	188	58
	5564_SNP2_SNP1_F	GATCGTAGAATTTGAAGCTCTA	5564_R	TTATTTTGTTGGCTTGAACC	191	53
m[5781]	5781_SNP_F	ATGAAGGCATTGGATCTAA	5781_R	TCTTTTAGGTTCTGGATTCTCG	203	53
m[6134]	6134_SNP1_Ins+SNP_R	TCAAAACTTCAATCCTTACTTGC	6134_F	ATCTCAGAGCGATTCAGAG	183—not obtained	Not obtained
	6134_Ins+SNP_SNP2_R	GGAGAAGGGAAAATCAAAACTT	6134_F	ATCTCAGAGCGATTCAGAG	196	58
	6134_SNP2_Ins+SNP_F	AAGTTTTGATTTTCCCTTCTTC	6134_R	CACAAGGATCATCAGCTATA	186	58
m[7853]	7853_SNP1_F	CAGAAAATGGAATGTTCTTGACA	7853_R	ACAATGGAAACTTTAACGAAT	324	53
	7853_SNP2_SNP1_F	GGAATGTTCTTGAGAGATCAT	7853_R	ACAATGGAAACTTTAACGAAT	316	50
	7853_SNP3_F	GGTGACAAATATGCCTCATG	7853_R	ACAATGGAAACTTTAACGAAT	284	58

**Table 3:** Summary of the number of intense bands observed in the tested DH line groups using the designed primer pairs. Results are presented only for primer pairs where the PCR product had the expected length.

Marker	Name of Spanning Primer	Name of Neighboring Primer	Intensive Bands Among 10 DH Lines Analyzed	
			Higher-Resistance DH Lines	Lower-Resistance DH Lines
m[12134]	12134_SNP_R	12134_F	3–5	0
m[12456]	12456_SNP_F	12456_R	4	0
m[12232]	12232_SNP_F	12232_R	5–6	1–2
m[4899]	4899_F	4899_R	7	0
m[5564]	5564_MNP_R	5564_F	7	0
	5564_SNP2_SNP1_F	5564_R	6	0
m[5781]	5781_SNP_F	5781_R	5	0
m[6134]	6134_SNP2_Ins+SNP_F	6134_R	7	0
m[7853]	7853_SNP1_F	7853_R	8	5
	7853_SNP2_SNP1_F	7853_R	8	5
	7853_SNP3_F	7853_R	8	5

**Table 4:** Detailed summary of primer pairs that yield a specific amplification product, considering their ability to differentiate plant groups with varying resistance levels and their sequencing-confirmed specificity.

Marker	Name of Spanning Primer	Name of Neighboring Primer	Differentiation of Plants with Contrasting Resistance (PCR)	Sequencing-Confirmed Detection of a Marker Allele
m[12134]	12134_SNP_R	12134_F	Weakly	Not sequenced
m[12456]	12456_SNP_F	12456_R	Yes	Yes
m[12232]	12232_SNP_F	12232_R	Yes	Yes
m[4899]	4899_F	4899_R	Yes	Yes
m[5564]	5564_MNP_R	5564_F	Yes	No
	5564_SNP2_SNP1_F	5564_R	Yes	Yes
m[5781]	5781_SNP_F	5781_R	Yes	Yes
m[6134]	6134_SNP2_Ins+SNP_F	6134_R	Yes	Not sequenced
m[7853]	7853_SNP1_F	7853_R	No	No
	7853_SNP2_SNP1_F	7853_R	No	No
	7853_SNP3_F	7853_R	No	No

**Table 5:** Doubled-haploid (DH) lines used in the study.

No.	Line ID	No.	Line ID
1.	DH2 0/21DH6720	11.	DH2 0/21DH6702
2.	DH2 0/21DH9455	12.	DH2 0/21DH6721
3.	DH2 0/21DH6762	13.	DH2 0/21DH6745
4.	DH2 0/21DH9440	14.	DH2 0/21DH6730
5.	DH21 0/21DH7414	15.	DH2 0/21DH6752
6.	DH21 0/21DH7433	16.	DH2 0/21DH9444
7.	DH2 0/21DH9525	17.	DH2 0/21DH6761
8.	DH22 0/21DH7477	18.	DH2 0/21DH9488
9.	DH22 0/21DH7481	19.	DH21 0/21DH7445
10.	DH20 0/21DH8950	20.	DH27 0/21DH7988

**Table 6:** DH lines used in the study for preliminary validation of primer pairs.

No.	Line ID	No.	Line ID
21.	OI21DH6612	31.	OI21DH9600
22.	OI21DH9593	32.	OI21DH9372
23.	OI21DH9389	33.	OI21DH5663
24.	OI21DH6404	34.	OI21DH6615
25.	OI21DH5829	35.	OI21DH9473
26.	OI21DH6572	36.	OIDH65563
27.	OI21DH6549	37.	OI21DH5684
28.	OI21DH6561	38.	OI21DH6542
29.	OI21DH9518	39.	OI21DH6544
30.	OI21DH6546	40.	OI21DH9536

**Table 7:** DH lines selected for Sanger sequencing based on the efficiency of identification of markers using PCR (DH line numbers according to Table 5).

Marker Name	DH Line that Generated Intense Bands	DH Line that Generated Weaker/No Bands
m[12134]	2	11
m[12456]	3	11
m[12232]	7	18
m[4899]	4	-
m[5564]	6	11
m[5781]	10	15
m[6134]	6	11
m[7853]	4	11

## Data Availability

The data presented in this study are available on request from the corresponding author.

## Supplementary Materials

The following supporting information can be downloaded at: https://www.mdpi.com/article/10.3390/ijms27146146/s1.
