# Supplementary material for: PCR Conditions for the Detection of Molecular Markers Associated with Blackleg (Leptosphaeria spp.) Resistance in Rapeseed (Brassica napus L.)
Source: Int J Mol Sci. 2026 Jul 9;27(14):6146. doi: 10.3390/ijms27146146 (PMC13409820; doi:10.3390/ijms27146146)
Supplement: Supplementary file 1 [file ijms-27-06146-s001.zip › ijms-4375306-supplementary.pdf]

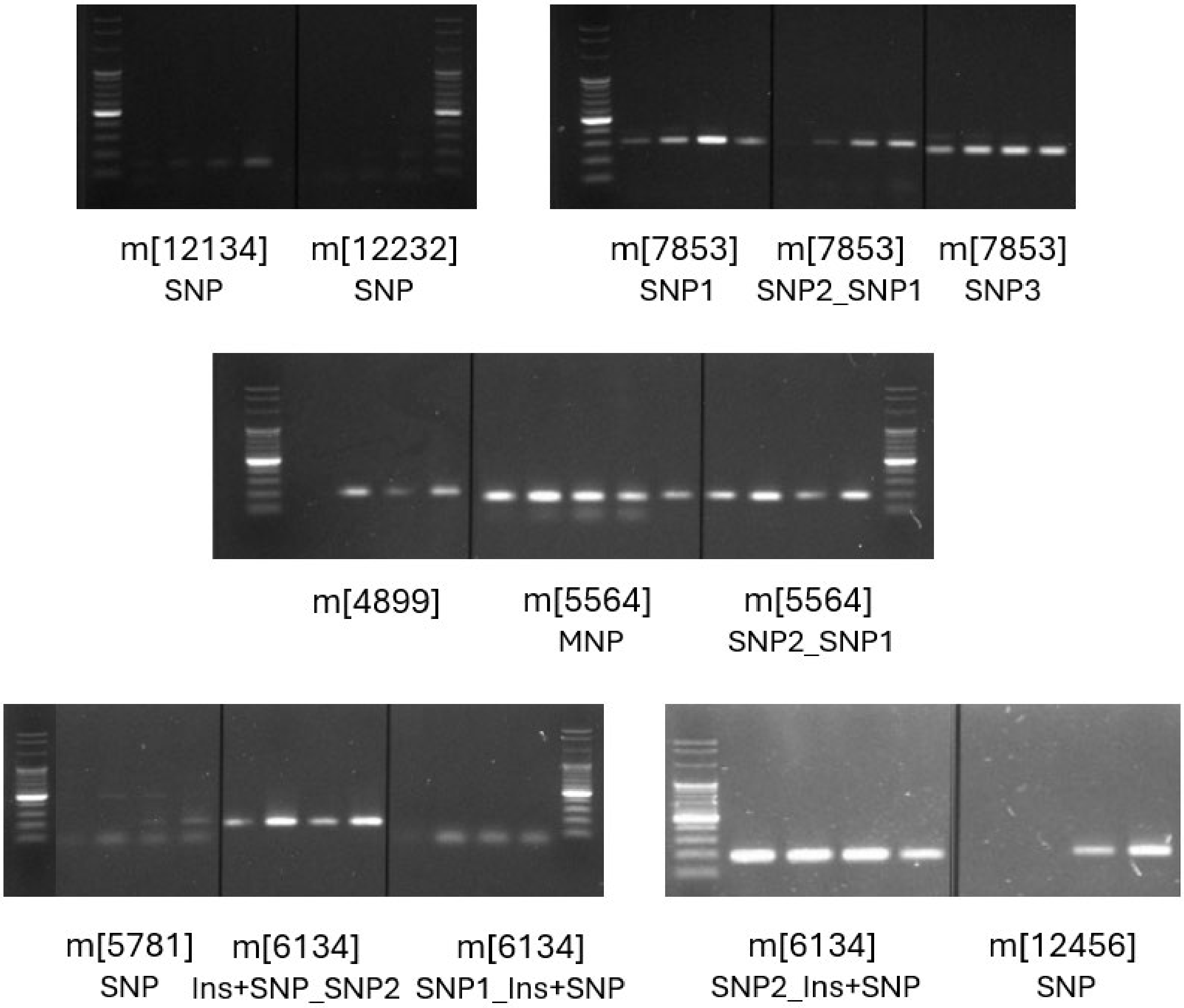

Supplementary Figure S1. Results of temperature gradient PCR with primers designed for identification of detected polymorphisms. Analyzed temperatures were (from the left): 59.2°C, 57.5°C, 53.2°C and 50.0°C. DNA molecular weight marker PerfectTM 100 bp DNA (EURx, Gdańsk, Poland) was used.

Supplementary Table S1. List of primer pairs used for the PCR amplification of marker *loci* (with flanking sequences) for further Sanger sequencing

Marker Name	Forward Primer Sequence	Reverse Primer Sequence	Expected Product Size [bp]	Annealing Temperature [°C]
m[12134	CGTCCTATCTTTTCCATAGAG	TTTGGAGTTGCGAAGAAC	272	62
m[12456	GCAGGTTCGATTTCCTAA	TGGAAAGCATTAAAGAAAA	302	62
m[12232	CGTTAAATATCATCCTTCTGTTC	CCAAATAAATAAGTATCGAAGTTCA	361	62
m[4899]	GGAGTACTTCACAGTTGAGG	AGACCCATTATCGCCTTATC	392	68
m[5564]	CTTGTAGTGTATAGGGGCTG	TTATTTTGTTGGCTTGAACC	374	65
m[5781]	AACTTCGTTAATAATTTTAATCAAA	TCTTTTAGGTTCTGGATTCTCG	711	62
m[6134]	ATCTCAGAGCGATTCAGAG	CACAAGGATCATCAGCTATA	360	65
m[7853]	TTTCTTTTCATATATTTTCTGATCT	ACAATGGAAACTTTAACGAAT	403	62

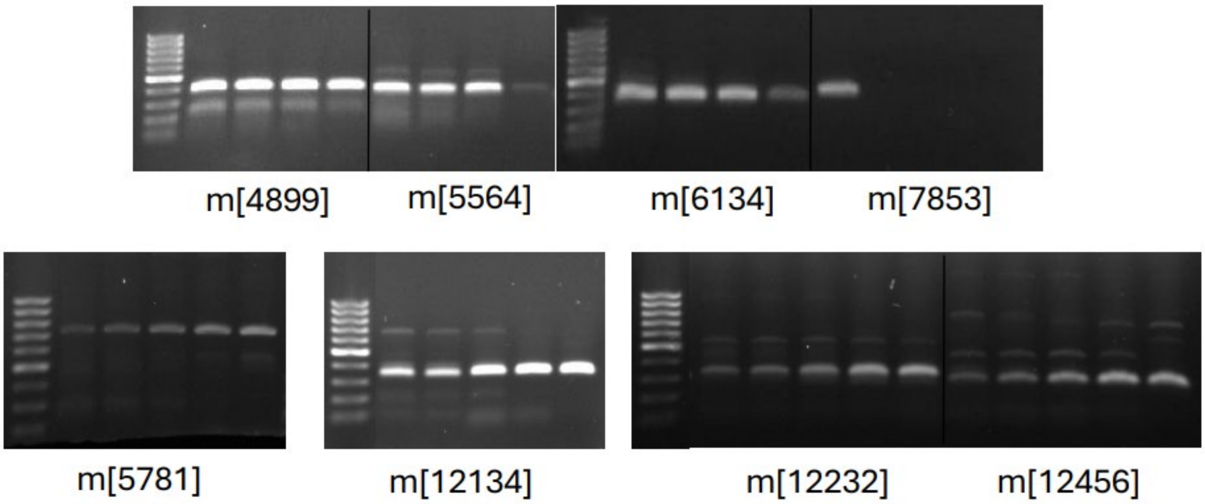

Supplemetary Figure S2. Results of temperature gradient PCR with primers designed for amplification of marker *loci* (with flanking sequences) for further Sanger sequencing. Analyzed temperatures were (from the left) (i) 62.0°C, 64.3°C, 65.7°C, 68.0°C for markers m[4899], m[5564], m[6134] and (ii) 50.0°C, 52.8°C, 55.5°C, 58.9°C, 61.6°C for markers m[5781], m[12134], m[12232], m[12456]. DNA molecular weight marker PerfectTM 100 bp DNA (EURx, Gdańsk, Poland) was used.

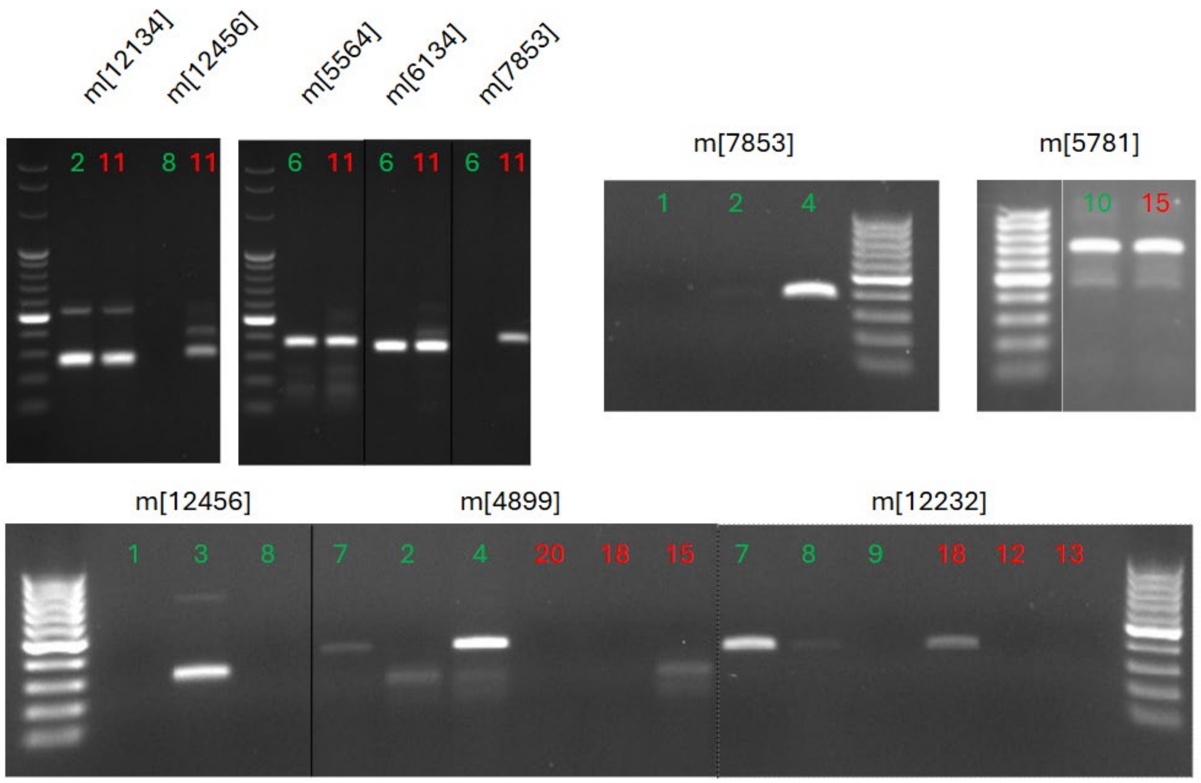

Supplementary Figure S3. Results of PCR amplification of markers using marker-spanning primer pairs for subsequent Sanger sequencing. Numbers highlighted in green and red indicate the DNA of DH lines used for amplification - those with higher and lower resistance, respectively.

6134_Ins+SNP_SNP2_R 5’-GGAGAAGGGAAAATCAAAACTT-3’

6134_F 5’-ATCTCAGAGCGATTCAGAG-3'

ATCTCAGAGCGATTCAGAGCTCTCTTCTCTCCGCAACTTGAAAGGCCCTGCACCTCGCAACGCTCT
AATCCTCGGTTAGTTTAGTCTCTCCTGCTCTCGAACCAAAAAAGTTTGTTACTTTGAGTAGAACGTTT
GAGATCAAAATAAAGGATTGATTTTTAAGTAAGTAAGGATTGAAGTTTTGATTTTCCCTTCTCCTGCA

6134_F (A07)	ATCTCAGAGCGATTCAGAG
BnaA07G0196400WE94_1	ATCTCAGAGCGATTCAGAG
BnA03g01279601135_17	ATCTCAGAGCGATTCAGAG
BnaC06G0196400ZY91_1	ATCTCAGAGCGATTCAGAG
chrA09g004818181_224	ATCTCGGAGCGATTCAGAG

Marker m[6134] (A07)	GTAAGTAAGGATTGAAGTTTTGATTTTCCCTTCTCCTGCA
BnaA07G0188900SL234_	TTAAGTAAGGATTGAAGTTTTGATTTTCCCTTCTCCTGCA
BnA03g01279601298_36	TTAAGTAAGGATTGAAGTTTTGATTTTCCCTTCTCGTGCA

Supplementary Figure S4. Analysis of the cause of non-specific PCR product formation above 500 bp for the primer pair 6134_F and 6134_Ins+SNP_SNP2_R. The sequences of the primers and their annealing sites on the *Brassica napus* DNA sequence are marked in blue and green. The given *B. napus* DNA sequence is the reference genome DNA sequence Darmor bzh 4.1, supplemented with the marker sequence at its occurrence site (underlined). The result of the BLAST analysis (https://yanglab.hzau.edu.cn/) of the sequence of both annealing sites for primer 6134_F and primer 6134_Ins+SNP_SNP2_R to marker m[6134] is also presented.

m[12456] intense band

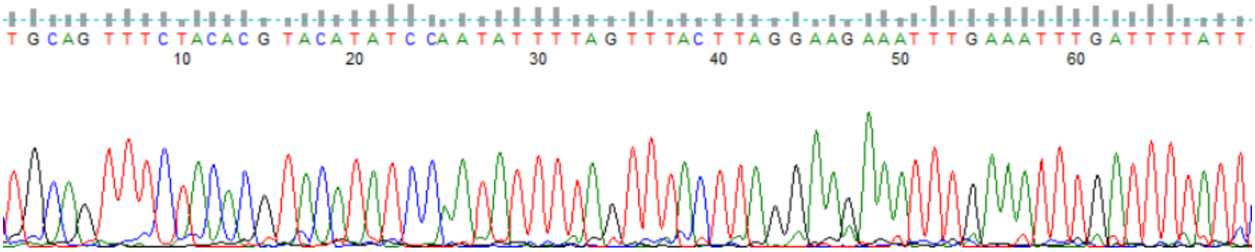

m[12456] no band

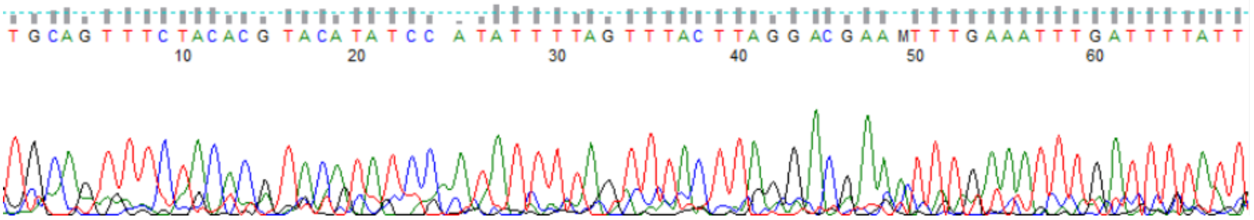

m[12232] intense band

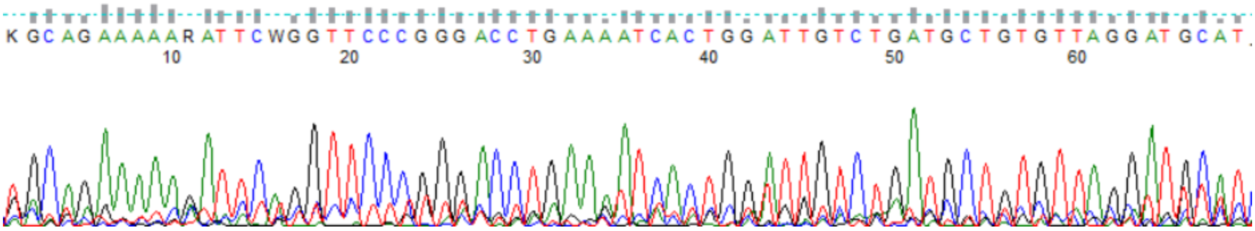

m[12232] weak band

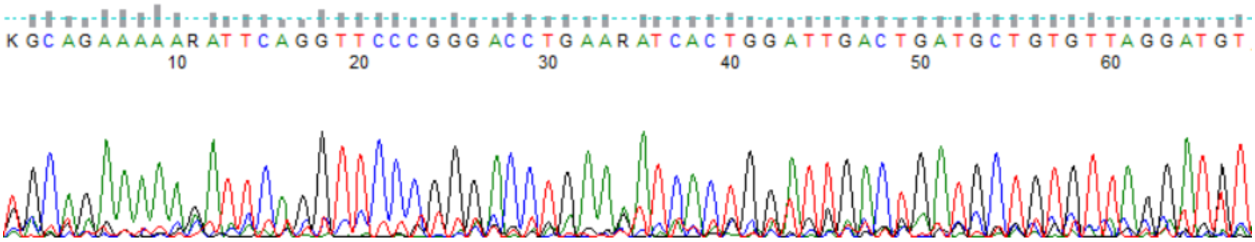

m[4899] intense band

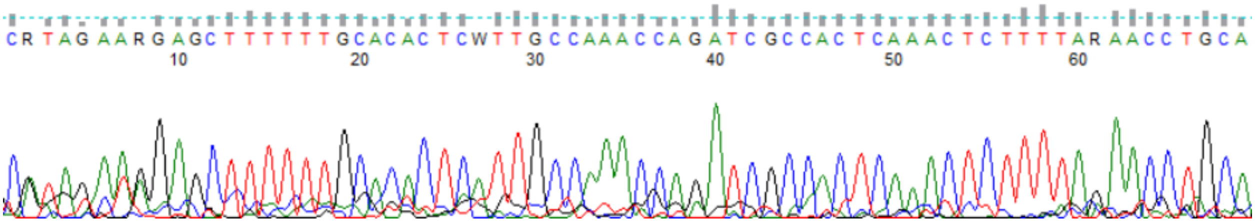

m[5564] intense band

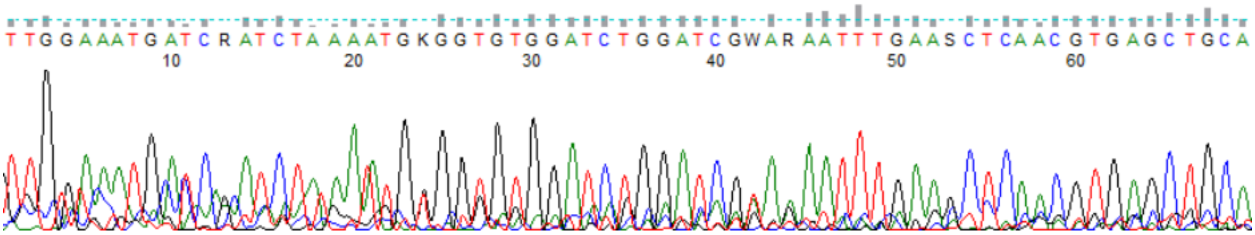

m[5564] no band

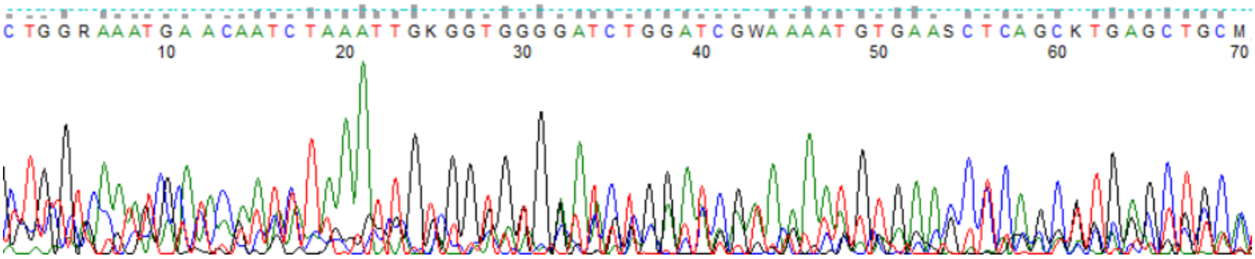

m[5781] **intense band**

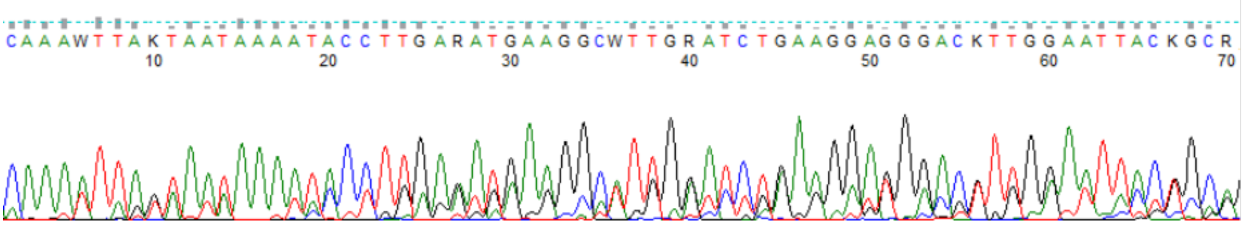

M[5781] **no band**

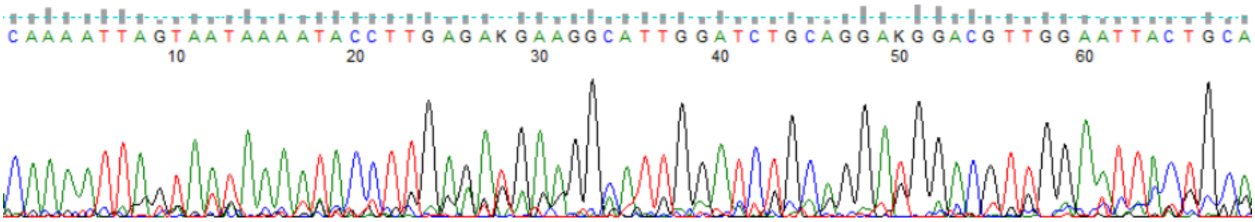

m[7853] **intense band**

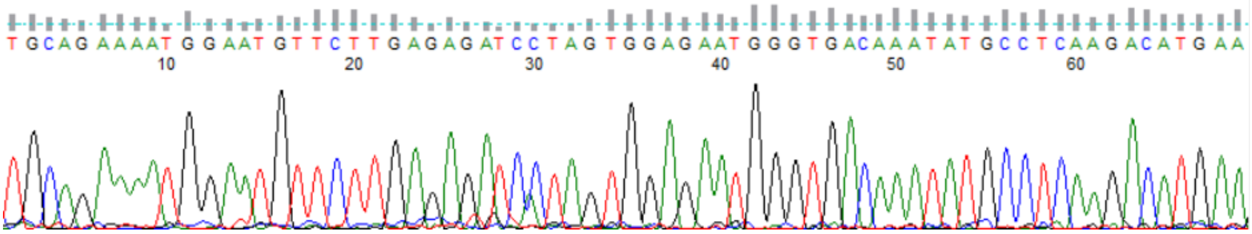

m[7853] **no band**

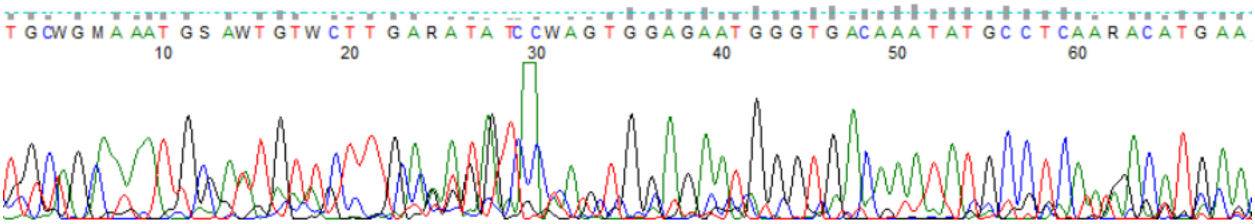

Supplementary Figure S5. Fluorograms obtained during Sanger sequencing of genotypes which yielded intense bands and weak/no bands during marker identification.

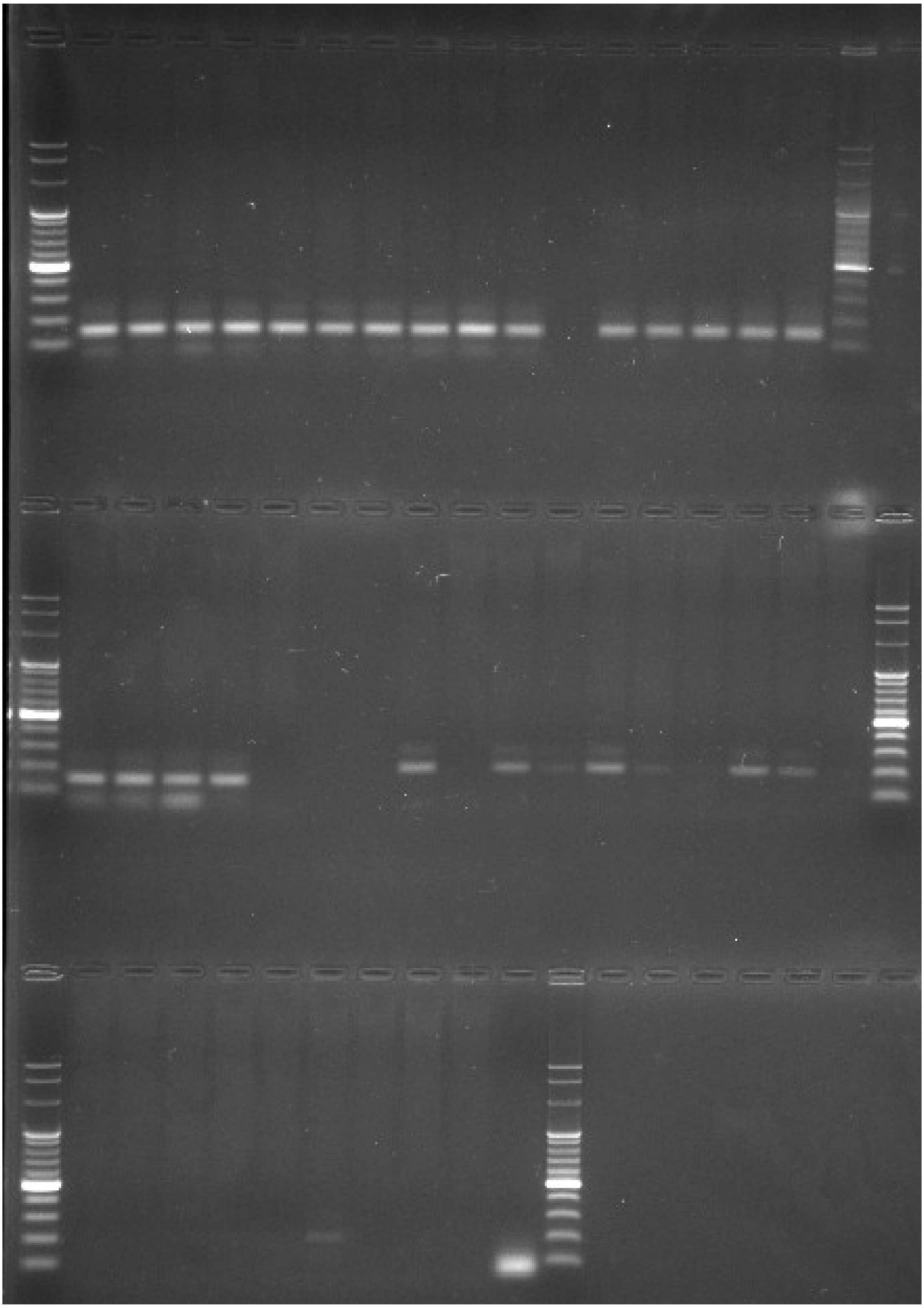

Supplementary Figure S6. Uncropped image of gel electrophoresis results of PCR products for identification of SNP-type markers: m[12134] (primers: 12134_SNP_R and 12134_F) and m[12456] (primers: 12456_SNP_F and 12456_R) in the DNA of rapeseed plants exhibiting higher (DH lines 1-10) and lower (DH lines 11-20) resistance to Leptosphaeria spp. Predicted PCR product sizes: 153 bp, and 215 bp. DNA molecular weight marker Perfect™ 100 bp DNA (EURx, Gdańsk, Poland).

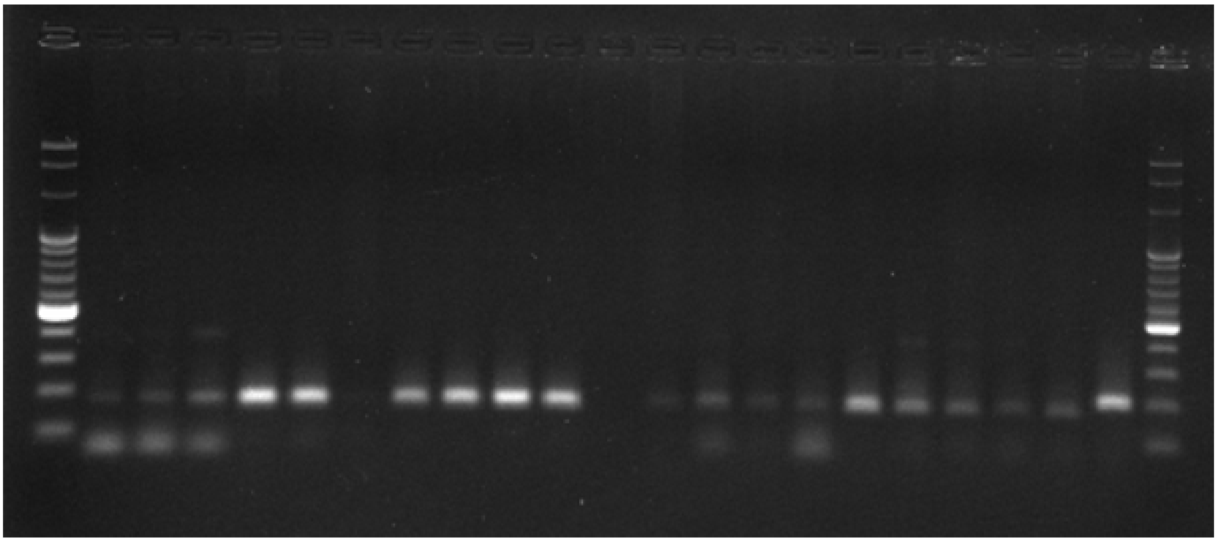

Supplementary Figure S7. Uncropped image of gel electrophoresis results of PCR products for identification of SNP-type marker: m[12232] (primers: 12232_SNP_F and 12232_R) in the DNA of rapeseed plants exhibiting higher (DH lines 1-10) and lower (DH lines 11-20) resistance to *Leptosphaeria* spp. Predicted PCR product size: 202 bp. DNA molecular weight marker Perfect^{TM} 100 bp DNA (EURx, Gdańsk, Poland).

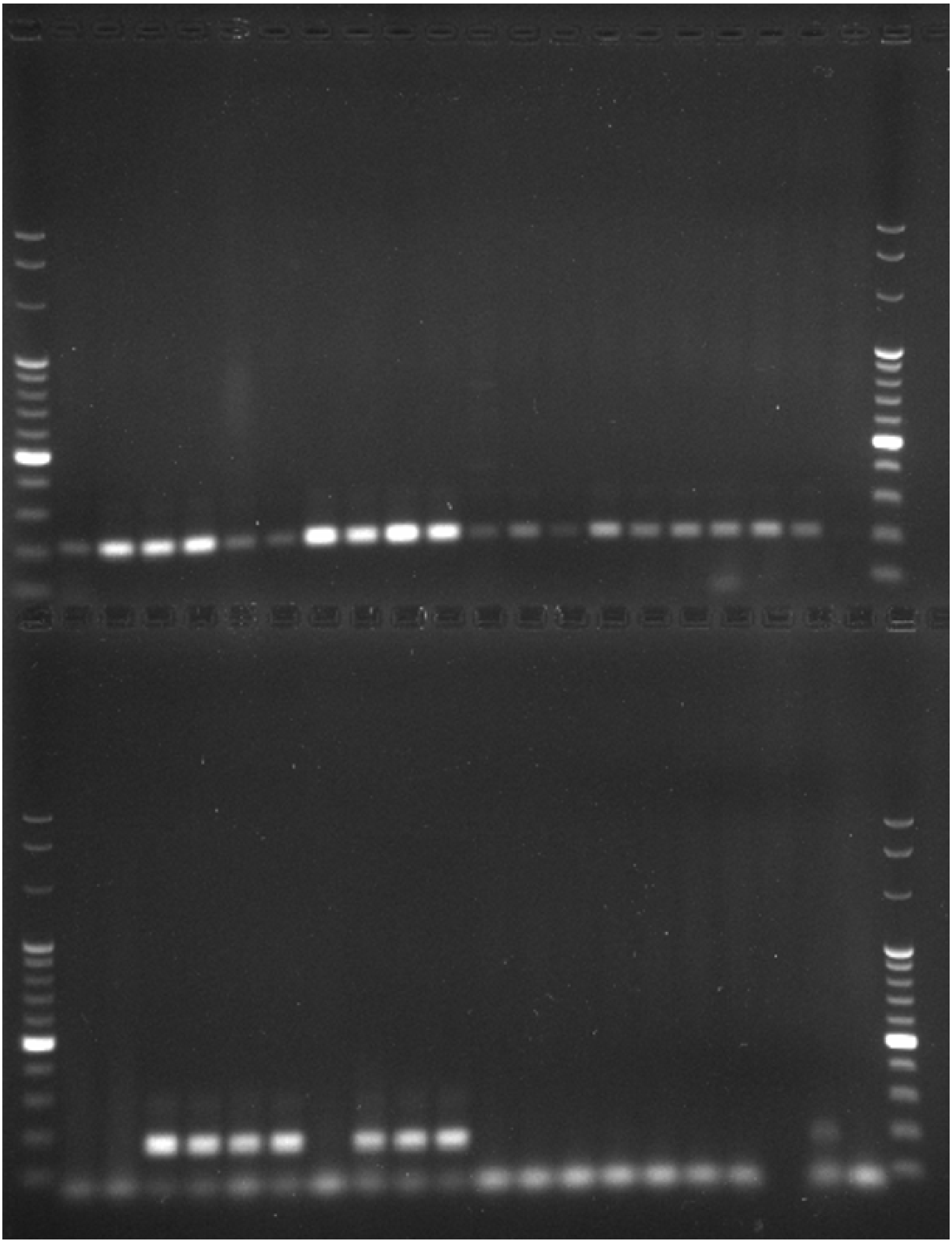

Supplementary Figure S8. Uncropped image of gel electrophoresis results of PCR products for identification of SilicoDArT-type markers: m[4899] (primers: 4899_F and 4899_R) and m[5564] (primers: 5564_MNP_R and 5564_F) in the DNA of rapeseed plants exhibiting higher (DH lines 1-10) and lower (DH lines 11-20) resistance to *Leptosphaeria* spp. Predicted PCR product sizes: 216 bp, and 188 bp. DNA molecular weight marker Perfect^{TM} 100 bp DNA (EURx, Gdańsk, Poland).

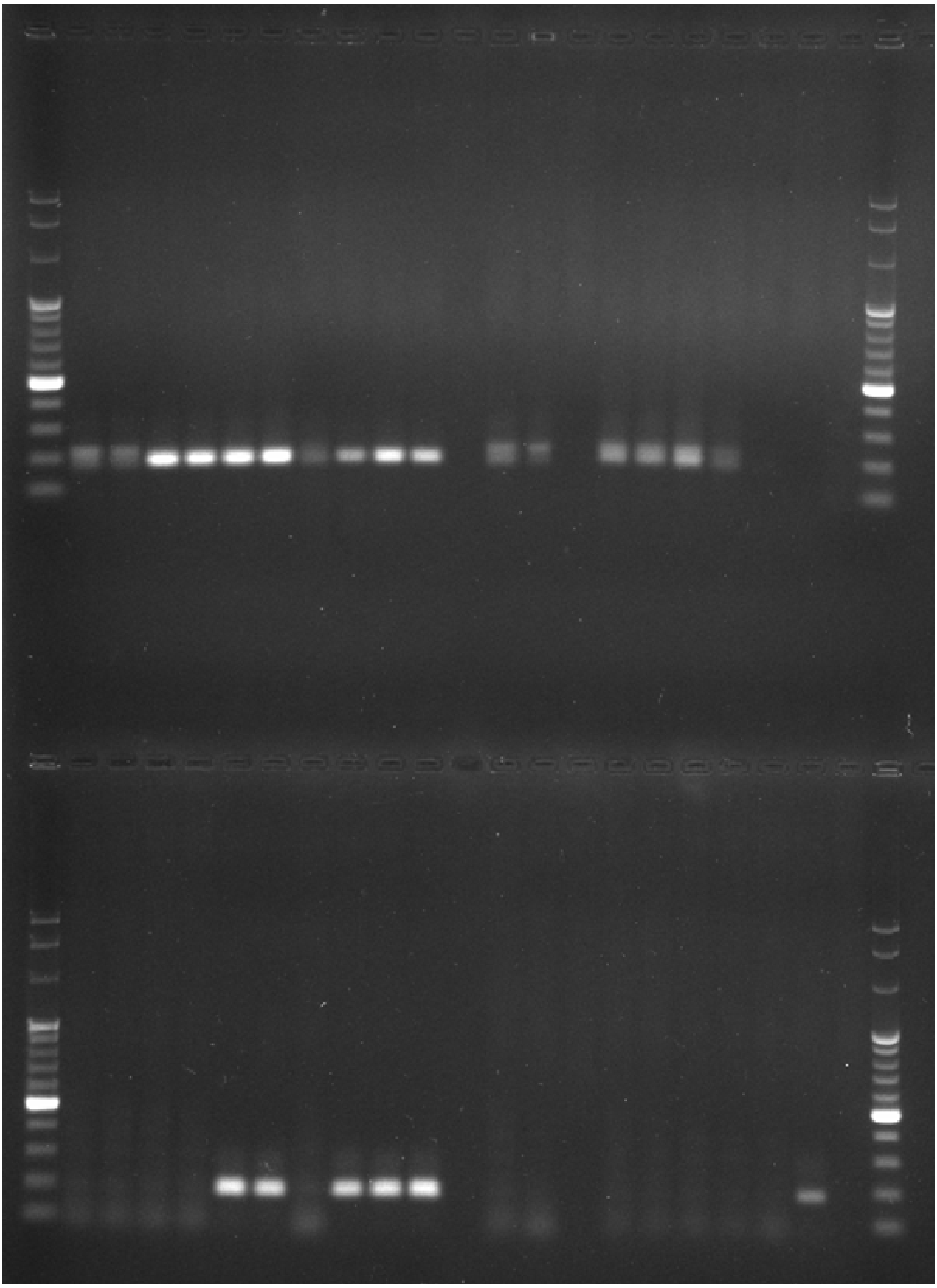

Supplementary Figure S9. Uncropped image of gel electrophoresis results of PCR products for identification of SilicoDArT-type markers: m[5564] (primers: 5564_SNP2_SNP1_F and 5564_R), m[5781] (primers: 5781_SNP_F and 5781_R) in the DNA of rapeseed plants exhibiting higher (DH lines 1-10) and lower (DH lines 11-20) resistance to Leptosphaeria spp. Predicted PCR product sizes: 191 bp, and 203 bp. DNA molecular weight marker Perfect^{TM} 100 bp DNA (EURx, Gdańsk, Poland).

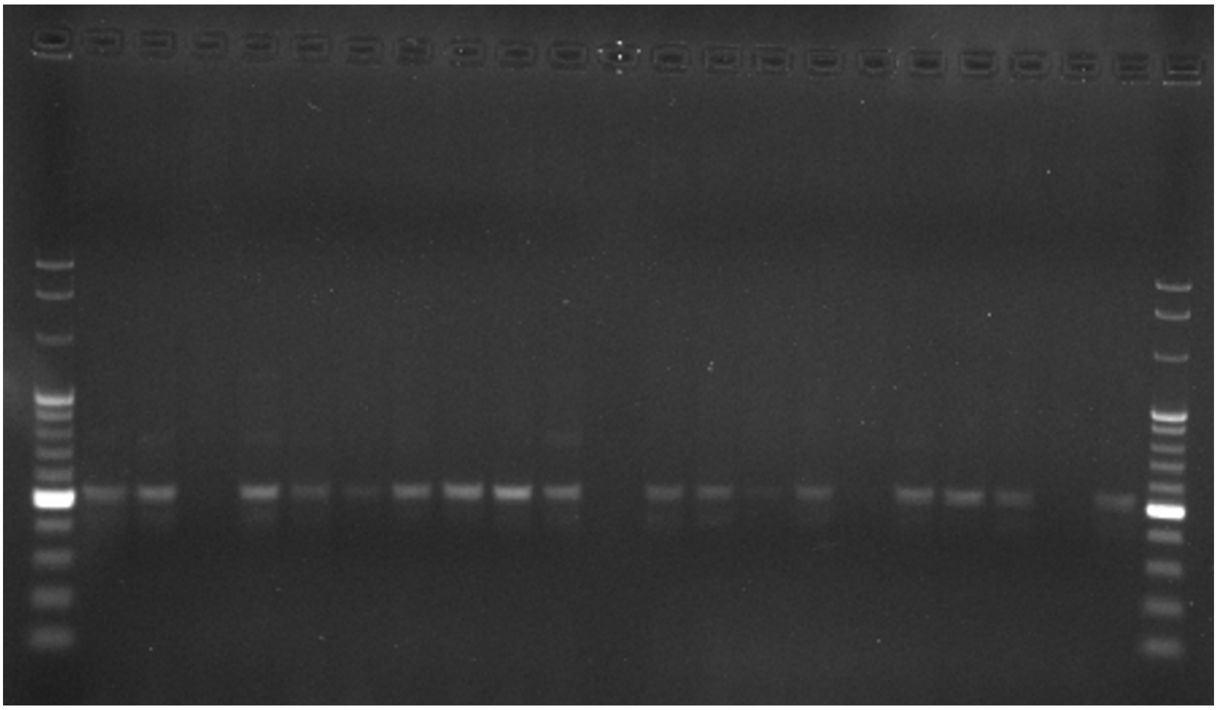

Supplementary Figure S10. Uncropped image of gel electrophoresis results of PCR products for identification of SilicoDArT-type marker: m[6134] (primers: 6134_Ins+SNP_SNP2_R and 6134_F) in the DNA of rapeseed plants exhibiting higher (DH lines 1-10) and lower (DH lines 11-20) resistance to *Leptosphaeria* spp. Predicted PCR product size: 196 bp. DNA molecular weight marker Perfect^{TM} 100 bp DNA (EURx, Gdańsk, Poland).

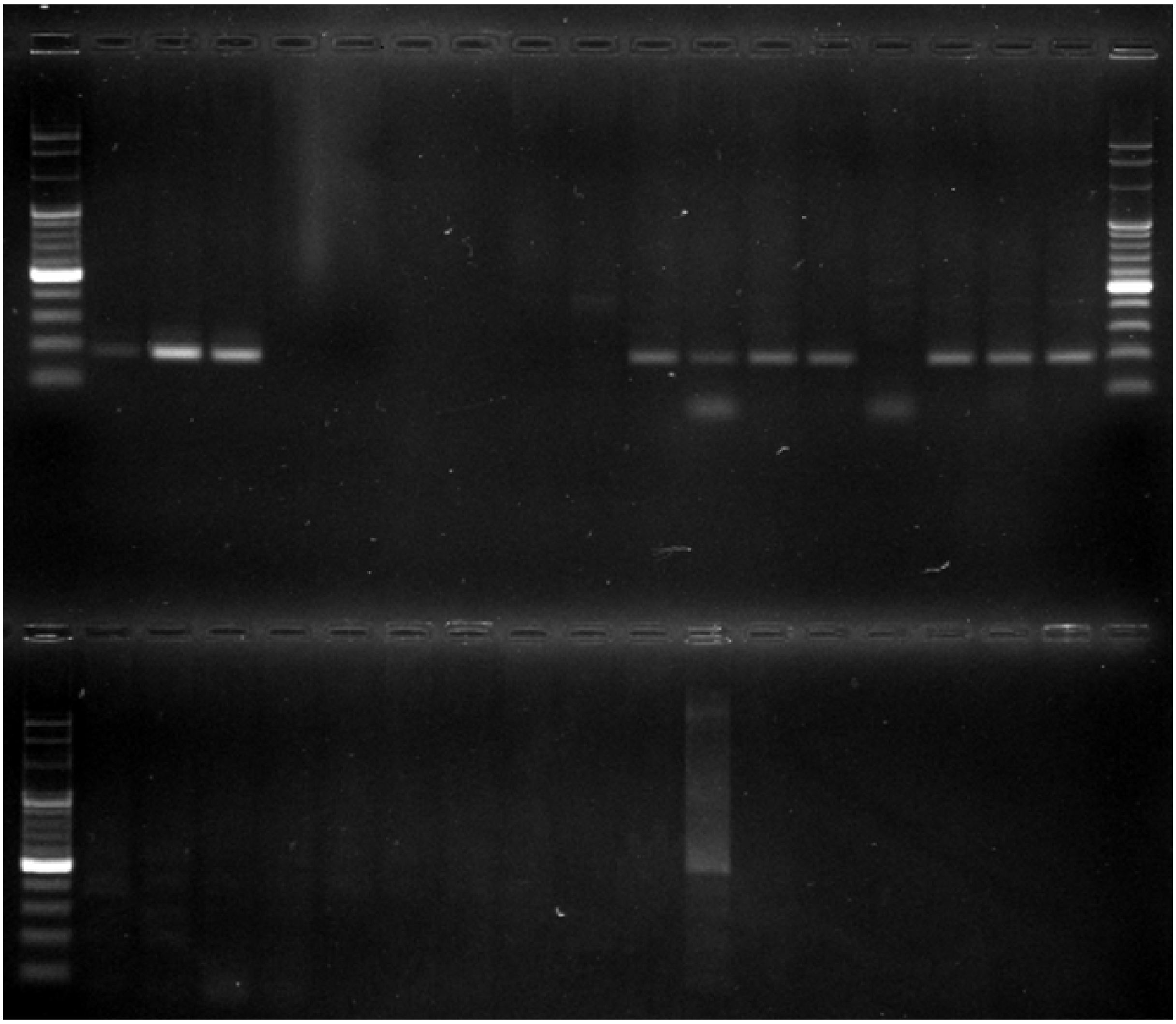

Supplementary Figure S11. Uncropped image of gel electrophoresis results of PCR products for identification of SilicoDArT-type marker: m[6134] (primers: 6134_SNP2_Ins+SNP_F and 6134_R) in the DNA of rapeseed plants exhibiting higher (DH lines 1-10) and lower (DH lines 11-20) resistance to *Leptosphaeria* spp. Predicted PCR product size: 186 bp. DNA molecular weight marker Perfect™ 100 bp DNA (EURx, Gdańsk, Poland).

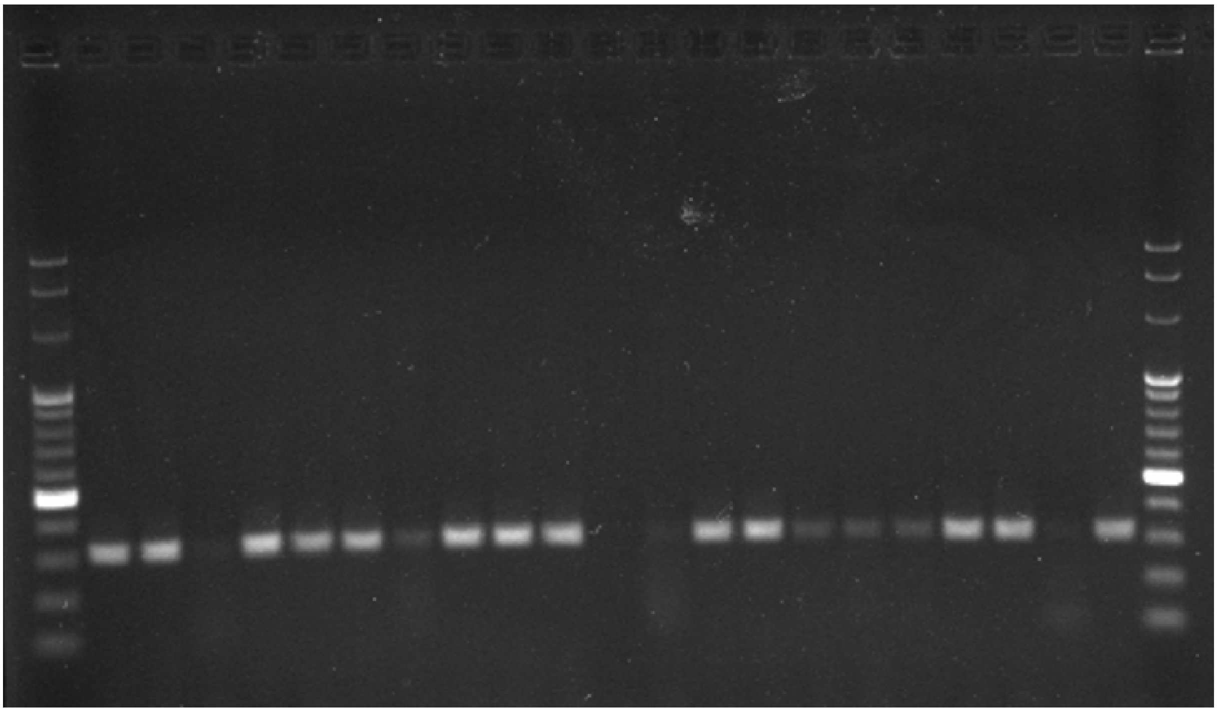

Supplementary Figure S12. Uncropped image of gel electrophoresis results of PCR products for identification of SilicoDArT-type marker: m[7853] (primers: 7853_SNP1_F and 7853_R) in the DNA of rapeseed plants exhibiting higher (DH lines 1-10) and lower (DH lines 11-20) resistance to *Leptosphaeria* spp. Predicted PCR product size: 324 bp. DNA molecular weight marker Perfect^{TM} 100 bp DNA (EURx, Gdańsk, Poland).

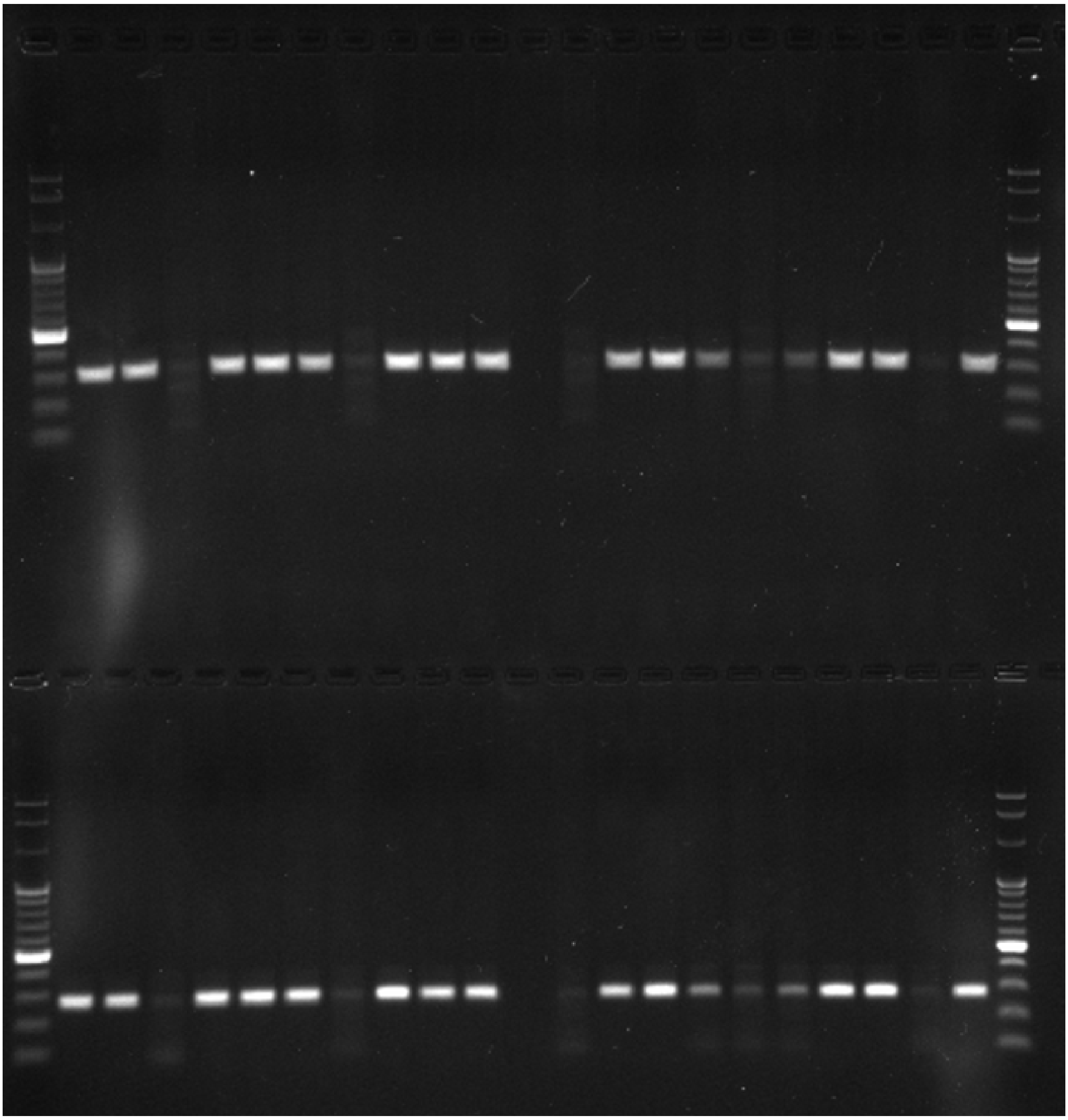

Supplementary Figure S13. Uncropped image of gel electrophoresis results of PCR products for identification of SilicoDArT-type marker: m[7853] (primers: 7853_SNP2_SNP1_F and 7853_R; 7853_SNP3_F and 7853_R) in the DNA of rapeseed plants exhibiting higher (DH lines 1-10) and lower (DH lines 11-20) resistance to *Leptosphaeria* spp. Predicted PCR product sizes: 316 bp, and 284 bp, respectively. DNA molecular weight marker Perfect^{TM} 100 bp DNA (EURx, Gdańsk, Poland).

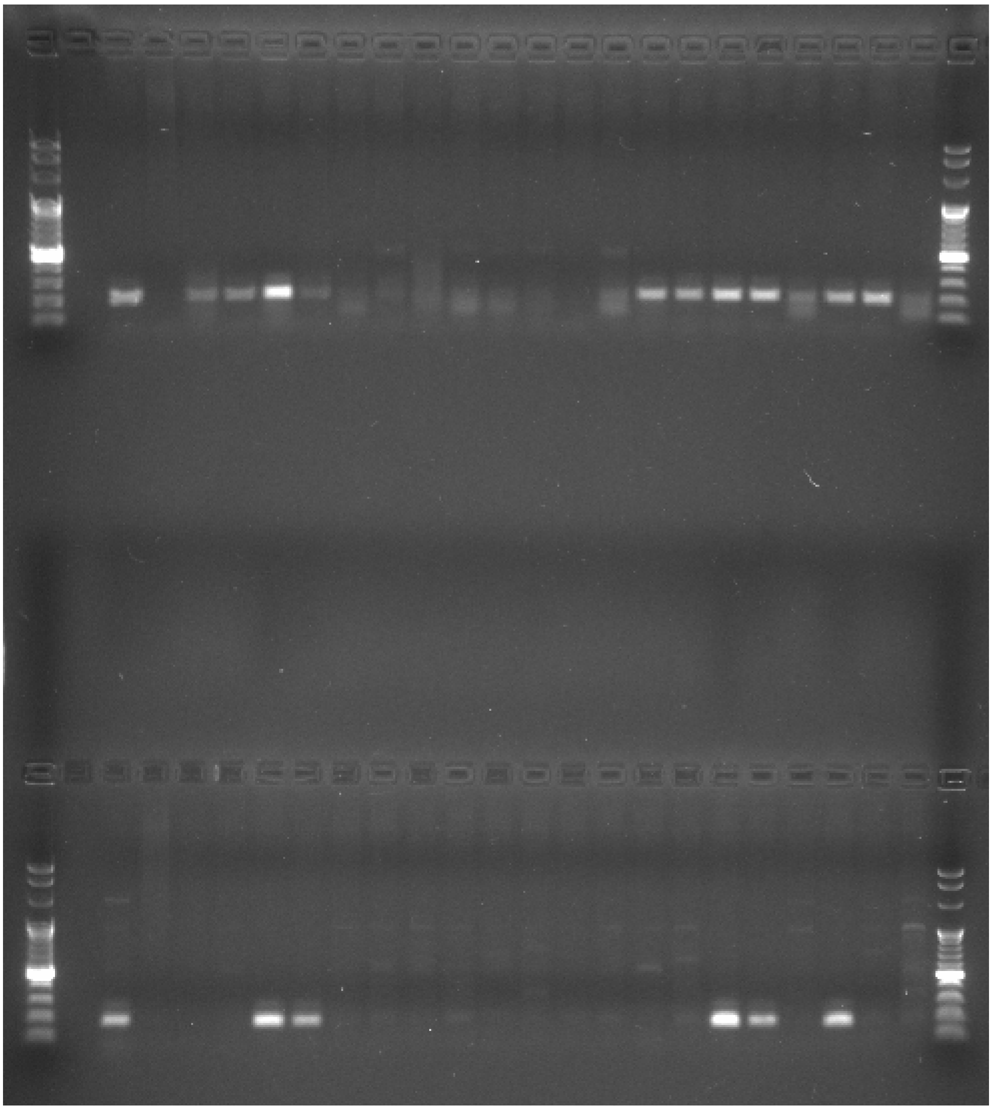

Supplementary Figure S14. Uncropped image of gel electrophoresis results of PCR comparative trial identifying SNP-type marker m[12456] (primers: 12456_SNP_F and 12456_R) and SilicoDArT-type marker m[6134] (primers: 6134_SNP2_Ins+SNP_F and 6134_R) in the DNA of 20 rapeseed DH lines. NTC: no template control, “+”: positive control (DNA of a DH line possessing the marker), "-": negative control (DNA of a DH line lacking the marker). Expected product sizes: 215 bp and 186 bp, respectively. DNA molecular weight marker PerfectTM 100 bp DNA (EURx, Gdańsk, Poland).

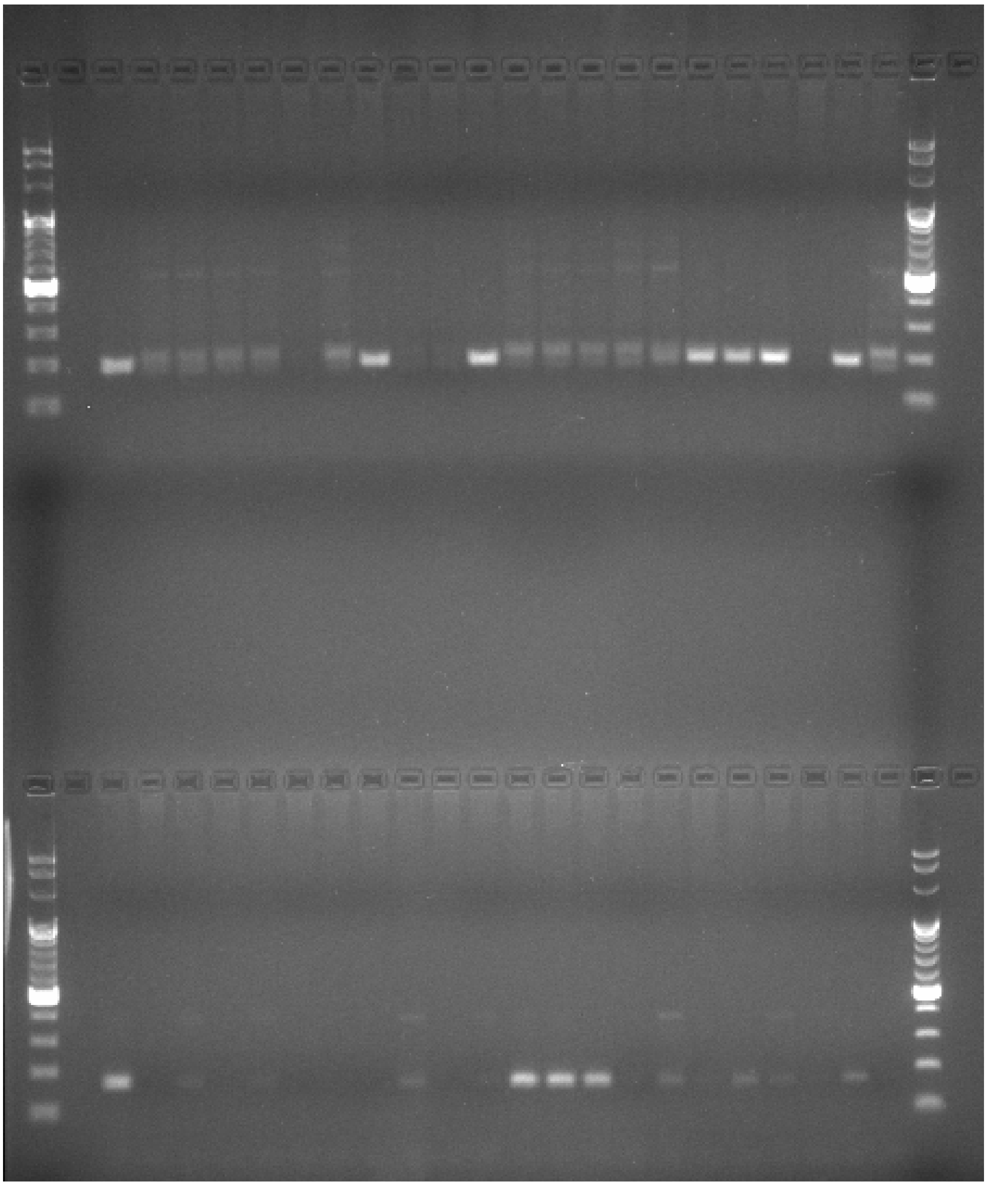

Supplementary Figure S15. Uncropped image of gel electrophoresis results of PCR comparative trial identifying SNP-type marker m[12232] (primers: 12232_SNP_F and 12232_R) and SilicoDArT-type marker m[5564] (primers: 5564_SNP2_SNP1_F and 5564_R) in the DNA of 20 rapeseed DH lines. NTC: no template control, “+”: positive control (DNA of a DH line possessing the marker), "-": negative control (DNA of a DH line lacking the marker). Expected product sizes: 202 bp and 191 bp, respectively. DNA molecular weight marker PerfectTM 100 bp DNA (EURx, Gdańsk, Poland).

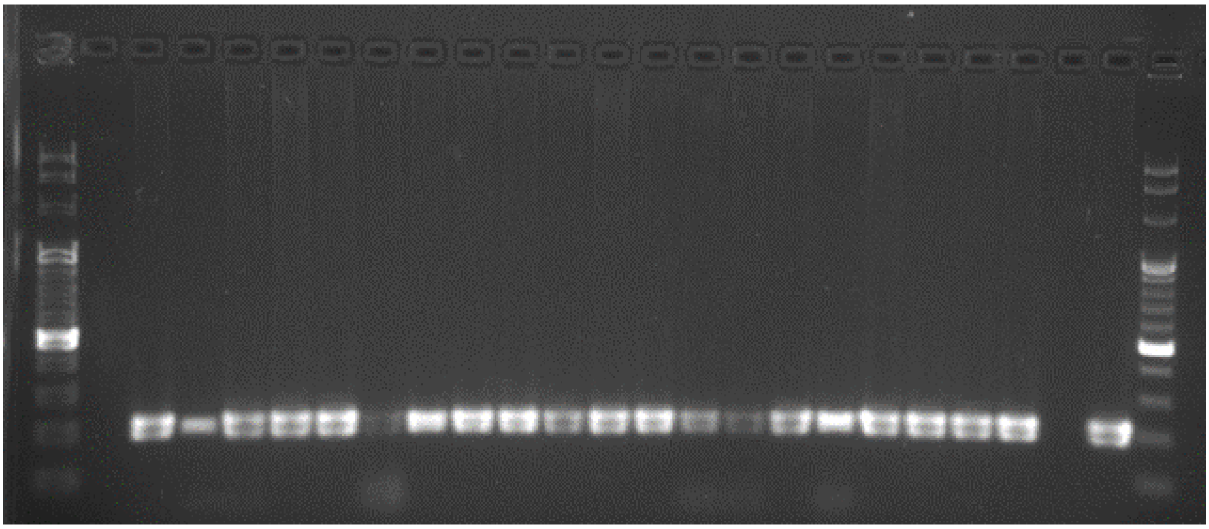

Supplementary Figure S16. Uncropped image of gel electrophoresis results of PCR comparative trial identifying SilicoDArT-type marker m[4899] (primers: 4899_F and 4899_R) in the DNA of 20 rapeseed DH lines. NTC: no template control, “+”: positive control (DNA of a DH line possessing the marker), "-": negative control (DNA of a DH line lacking the marker). Expected product size: 216 bp. DNA molecular weight marker PerfectTM 100 bp DNA (EURx, Gdańsk, Poland).

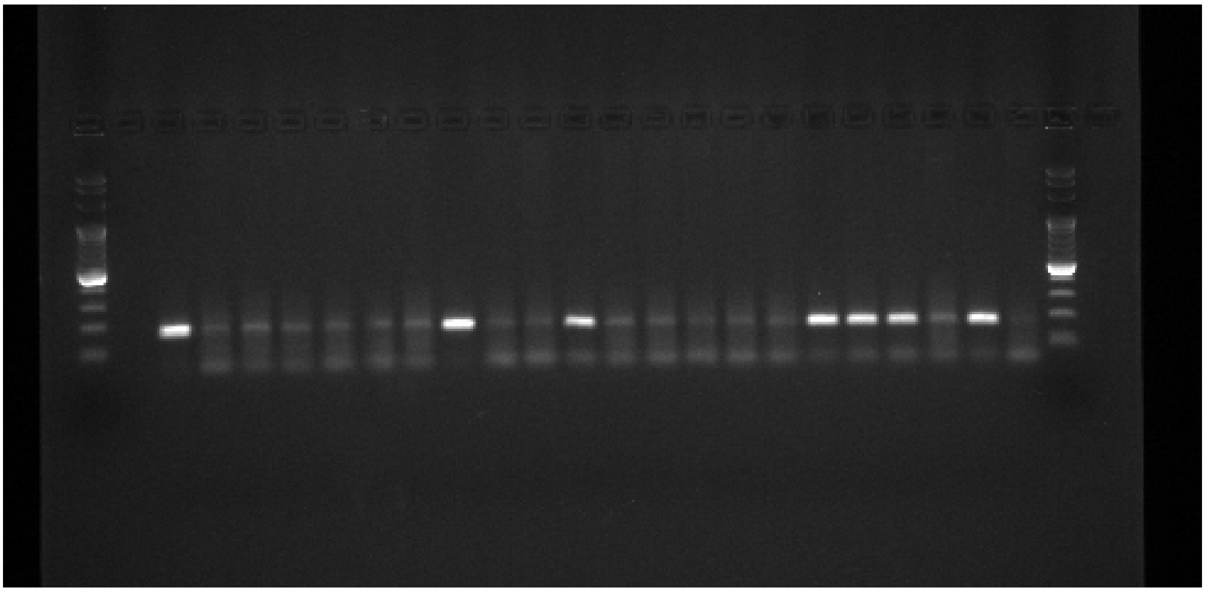

Supplementary Figure S17. Uncropped image of gel electrophoresis results of PCR comparative trial identifying SilicoDArT-type marker m[5564] (primers: 5564_MNP_R and 5564_F) in the DNA of 20 rapeseed DH lines. NTC: no template control, "+": positive control (DNA of a DH line possessing the marker), "-": negative control (DNA of a DH line lacking the marker). Expected product size: 188 bp. DNA molecular weight marker PerfectTM 100 bp DNA (EURx, Gdańsk, Poland).

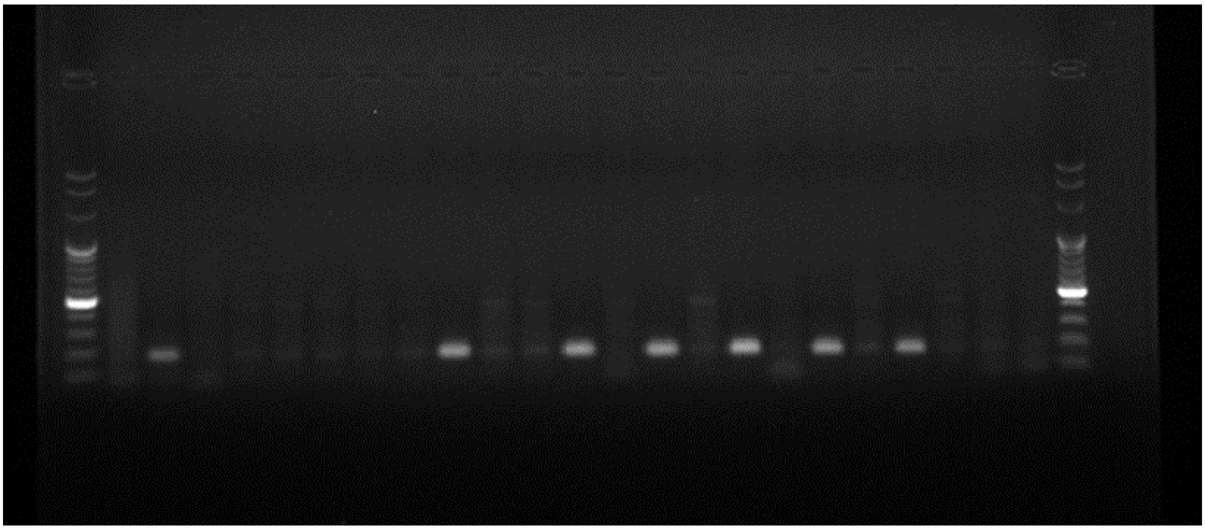

Supplementary Figure S18. Uncropped image of gel electrophoresis results of PCR comparative trial identifying SilicoDArT-type marker m[5781] (primers: 5781_SNP_F and 5781_R) in the DNA of 20 rapeseed DH lines. NTC: no template control, "+": positive control (DNA of a DH line possessing the marker), "-": negative control (DNA of a DH line lacking the marker). Expected product size: 203 bp. DNA molecular weight marker PerfectTM 100 bp DNA (EURx, Gdańsk, Poland).
